# A novel stress-inducible CmtR-ESX3-Zn^2+^ regulatory pathway essential for survival of *Mycobacterium bovis* under oxidative stress

**DOI:** 10.1074/jbc.RA120.013017

**Published:** 2020-10-08

**Authors:** Xiaohui Li, Liu Chen, Jingjing Liao, Jiechen Hui, Weihui Li, Zheng-Guo He

**Affiliations:** 1 College of Life Science and Technology, Huazhong Agricultural University, Wuhan, China; 2 State Key Laboratory for Conservation and Utilization of Subtropical Agro-bioresources, College of Life Science and Technology, Guangxi University, Nanning, China

**Keywords:** Mycobacterium bovis, oxidative stress, ESX-3, zinc ion, CmtR, gene regulation, gene transcription, bacterial genetics, bacterial pathogenesis, microbiology, zinc, mycobacterium

## Abstract

Reactive oxygen species (ROS) are an unavoidable host environmental cue for intracellular pathogens such as *Mycobacterium tuberculosis* and *Mycobacterium bovis*; however, the signaling pathway in mycobacteria for sensing and responding to environmental stress remains largely unclear. Here, we characterize a novel CmtR-Zur-ESX3-Zn^2+^ regulatory pathway in *M. bovis* that aids mycobacterial survival under oxidative stress. We demonstrate that CmtR functions as a novel redox sensor and that its expression can be significantly induced under H_2_O_2_ stress. CmtR can physically interact with the negative regulator Zur and de-represses the expression of the *esx*-3 operon, which leads to Zn^2+^ accumulation and promotion of reactive oxygen species detoxication in mycobacterial cells. Zn^2+^ can also act as an effector molecule of the CmtR regulator, using which the latter can de-repress its own expression for further inducing bacterial antioxidant adaptation. Consistently, CmtR can induce the expression of EsxH, a component of *esx*-3 operon involved in Zn^2+^ transportation that has been reported earlier, and inhibit phagosome maturation in macrophages. Lastly, CmtR significantly contributes to bacterial survival in macrophages and in the lungs of infected mice. Our findings reveal the existence of an antioxidant regulatory pathway in mycobacteria and provide novel information on stress-triggered gene regulation and its association with host–pathogen interaction.

Gene expression at accurate time points is of critical importance for the rapid adaptation of pathogens to harsh environments. Reactive oxygen species (ROS) are an unavoidable environmental stimuli for nearly all organisms. This particularly holds true for intracellular pathogens such as *Mycobacterium tuberculosis* (Mtb) and *Mycobacterium bovis*. During infection, the intracellular pathogen is exposed to a variety of host-mediated stresses, in which ROS is one of the primary antimicrobial agents produced by phagocyte oxidase in macrophages (1–4). Excess ROS can induce oxidative stress and, therefore, control bacterial infection by damaging essential cellular components such as proteins, lipids, and nucleic acids in bacteria (5). *M. tuberculosis* is one of the most persistent intracellular pathogens and possesses unique mechanisms to combat oxidative stress for survival in host cells. For example, Mtb cells have a thick wall (6) and contain mycothiol at millimolar concentrations (7, 8); in particular, Mtb encodes multiple protective enzymes such as catalase (KatG) (9), superoxide dismutases (10), and peroxynitrite reductase complex (11, 12). However, the molecular mechanism and the signaling pathways by which mycobacteria adaptively promote antioxidant regulation are yet to be elucidated.

Specific regulatory sensors have evolved in certain bacteria to detect different environmental cues, such as the presence or absence of oxygen or ROS (13). By binding different cofactors, these sensors can convert oxidative signals into regulatory outputs and further trigger bacterial adaptation to stress (13). For example, OxyR is a well-characterized redox sensor in *Escherichia coli* (14, 15). The cysteine residues in it can be oxidized to form an intramolecular disulfide bond and promote the *katG* expression (16). By sensing and responding to the presence of organic hydroperoxides, *Bacillus subtilis* OhrR regulates the expression of organic hydroperoxide reductase (*ohrA*) to support bacterial survival in toxic environments (17). In mycobacterial species, only redox-sensing regulators have been characterized clearly, of which MosR is a redox-dependent transcription factor similar to *B. subtilis* OhrR (18). In addition, OxyS has also been characterized as a redox sensor that regulates the expression of *katG* (19).

In recent times, a growing body of evidence has suggested that metal ions, including iron, manganese, and even zinc ions, can function as essential structural or catalytic cofactors for activating antioxidant enzymes in certain bacteria (20–24). ESX-3 is conserved in several mycobacterial species and is one of the five type-VII secretion systems for exporting proteins linked to tuberculosis pathogenesis (25, 26). EsxH, which is one of the components encoded by the *esx*-3 operon, can target the host endosomal sorting complex to impair phagosome maturation and the recognition of Mtb-infected cells (27, 28). The EsxG-EsxH heterodimer can induce the host immunopathologic response and improve bacterial survival during infection (29, 30). Notably, the expression of the *esx-3* operon is regulated by the zinc uptake repressor (Zur) and the iron-dependent transcriptional repressor (IdeR) to maintain zinc and iron homeostasis, respectively, in *M. tuberculosis* (31–33). The EsxG-EsxH complex contains a specific Zn^2+^ binding site formed of a cluster of histidine residues in EsxH (34) and has been linked to adaptations of *M. tuberculosis* under low zinc-concentration environments. The expression and secretion of the EsxG-EsxH complex have been observed to significantly affect the interaction between *M. tuberculosis* and macrophages, as well as bacterial survival in host cells (35). However, the roles of Zn^2+^ in mycobacterial antioxidant defense and those of ESX-3 in the maintenance of metal homeostasis remain unclear. The mechanism by which a redox sensor is potentially linked to the expression of the *esx-3* operon, if it is linked at all, and its effect on bacterial survival under oxidative stress as well as within the host remain unexplored.

In this study, we report that CmtR, usually regarded as a Cd/Pb sensor (36–38), is a novel redox sensor in *M. bovis* that is essential for mycobacterial growth under oxidative stress. Upon sensing an oxidative stress signal, CmtR can trigger the expression of the *esx-3* operon by physically interacting with Zur to de-repress its activity, which affects Zn^2+^ homeostasis in bacterial cells and enhances bacterial antioxidant adaptation. Meanwhile, Zn^2+^ can also act as an effector molecule of the CmtR regulator to further promote the antioxidant regulation pathway. Lastly, CmtR was observed to significantly contribute to bacterial survival during infection. Our findings indicate the existence of a novel oxidative stress-triggered signaling circuit and provide new insights into mycobacterial adaptation to the host environment during infection.

## Results

### CmtR enhances the resistance of M. bovis BCG to H_2_O_2_

**Figure 1.**
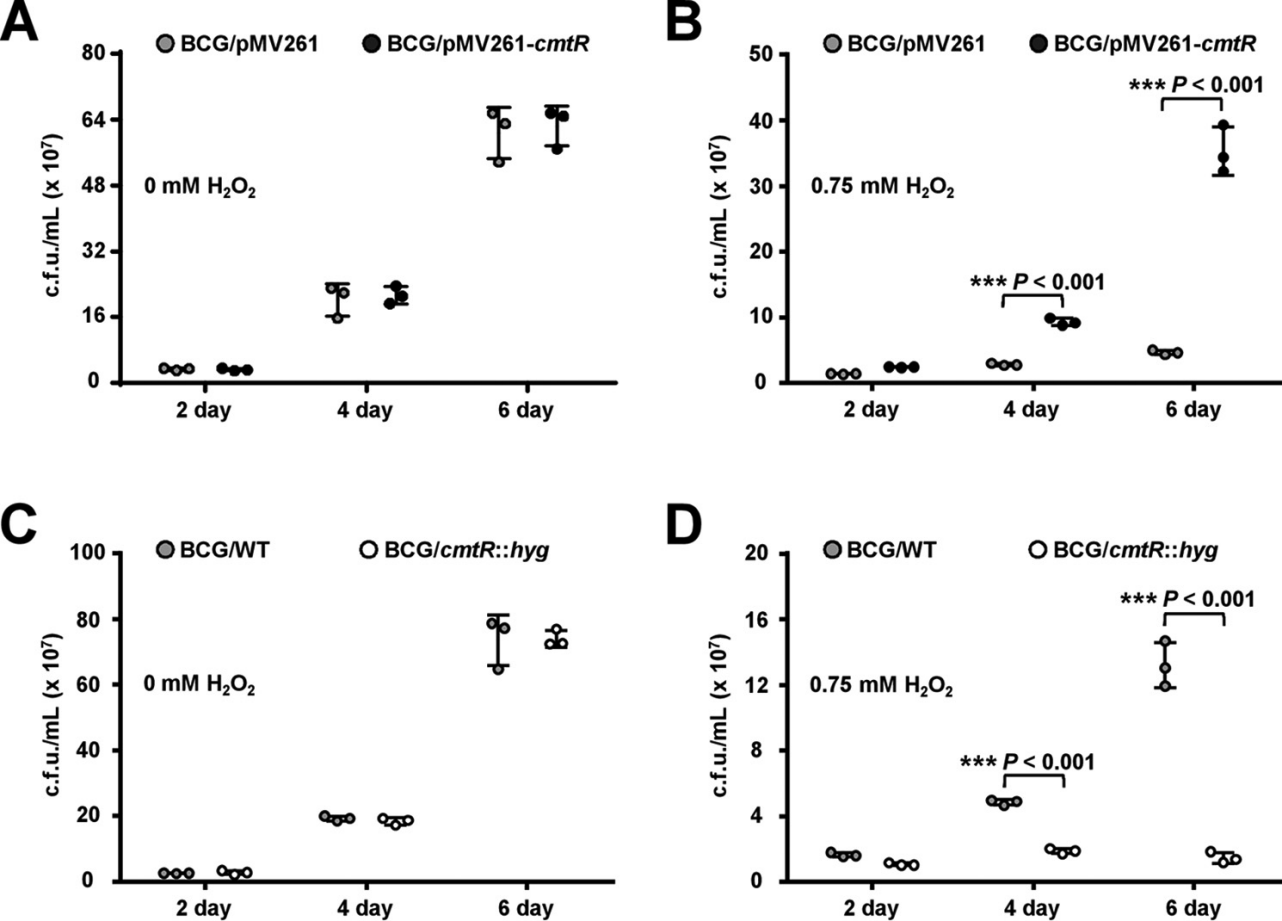
**Assays for the regulatory effect of CmtR on H_2_O_2_ resistance of *M. bovis* BCG strains.** *A* and *B*, comparative assays for the growth difference between BCG/pMV261 (control) and BCG/pMV261-cmtR (cmtR overexpression) strains in 7H9 medium (*A*) and in medium supplemented with 0.75 mm H_2_O_2_ (*B*). *C* and *D*, comparative assays for the growth difference between BCG/WT (control) and BCG/cmtR::hyg (cmtR-deleted) strains grown in 7H9 medium (*C*) and in medium supplemented with 0.75 mm H_2_O_2_ (*D*). *Error bars* represent the S.D. from three biological experiments. The *P*-values of the data were calculated by unpaired two-tailed Student's *t* test using GraphPad Prism 7. *Asterisks* denote significant difference (***, *P* < 0.001, two-tailed Student's *t* test) between two groups.

In a previous screening of the transcriptional factor library of *M. tuberculosis*, we preliminarily characterized CmtR as a potential regulator that contributes to H_2_O_2_ resistance of *M. bovis* BCG (Fig. S1*A*). In this study, we further determined the effects of the varying expression levels of *cmtR* on mycobacterial growth under H_2_O_2_ stress. As shown in Fig. 1, when comparing the growth of two mycobacterial strains treated with 0.75 mm H_2_O_2_, the bacterial counts of the *cmtR* overexpressing *M. bovis* were significantly higher than those of the WT strain at 4 and 6 days (Fig. 1*B*), which indicates that *cmtR* overexpression enhanced the resistance of *M. bovis* to H_2_O_2_. In contrast, the *cmtR*-deleted strain was more sensitive to H_2_O_2_ than the WT strain (Fig. 1*D*). In the absence of H_2_O_2_ stress, no obvious growth difference was observed between the WT strain and the overexpressing or deletion strains (Fig. 1, *A* and *C*). These results indicate that *cmtR* expression enhances the resistance of *M. bovis* BCG to H_2_O_2_.

### H_2_O_2_ decreases the DNA-binding ability of CmtR both in vitro and in M. bovis

**Figure 2.**
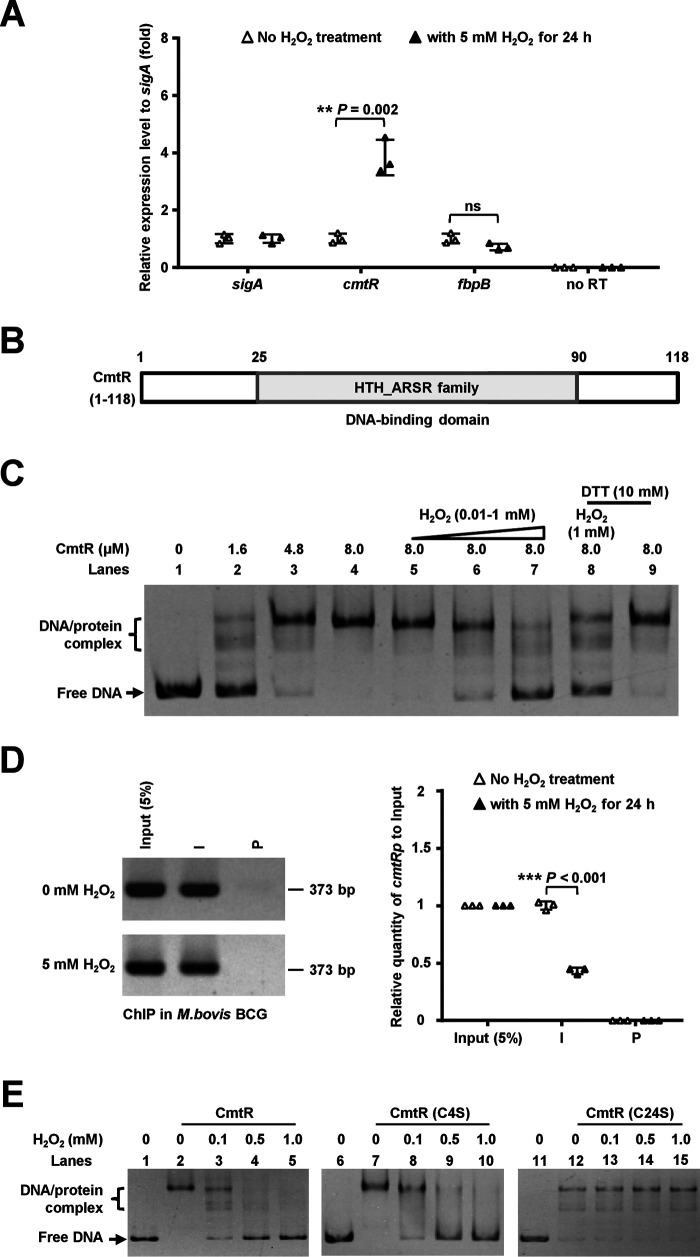
**Assays for studying the effects of H_2_O_2_ on the DNA-binding ability of CmtR.** *A*, qRT-PCR assays for *cmtR* expression in *M. bovis* BCG strains under 5 mm H_2_O_2_ stress. *B*, domain assay for *M. tuberculosis* CmtR by searching the Conserved Domain Database (CDD database) in the NCBI website. *C*, EMSA for studying the effects of H_2_O_2_ and DTT on the DNA-binding activity of CmtR. The CmtR promoter DNA substrate was co-incubated with increasing concentrations of CmtR (1.6-8.0 μm) (*lanes 2*–*4*) in the presence of H_2_O_2_ (*lanes 5–7* and *lane 8*) or DTT (*lanes 8* and *9*). *D*, ChIP assays for studying the effect of H_2_O_2_ on the intracellular DNA-binding activity of CmtR in *M. bovis* BCG. The *Input (5%)* indicated that the supernatant of disrupted cells was diluted to 5% and used as the PCR template. ChIP using preimmune (*P*) or immune (*I*) sera raised against CmtR. The expression levels were quantified using qPCR (*right panel*). The *P*-values of the data were calculated by two-tailed Student's *t* test using GraphPad Prism 7. *Asterisks* represent significant difference (**, *P* < 0.01; ***, *P* < 0.001; *ns*, not significant, two-tailed Student's *t* test) between two groups. *E*, assays for studying the effect of H_2_O_2_ on the DNA-binding activity of mutant CmtR-C24S proteins. The protein concentration was 3.2 μm, and the different concentrations of H_2_O_2_ are indicated at the *top of the panels*. The protein/DNA complexes and free DNA are indicated by *arrows* on the *left side* of the panels.

To elucidate the CmtR-triggered signaling pathway that regulates mycobacterial resistance to oxidative stress, we first determined whether the redox reagent, H_2_O_2_, induces *cmtR* expression in *M. bovis* BCG. To this end, we performed a qRT-PCR assay to evaluate the differential expression of *M. bovis cmtR* in the presence or absence of H_2_O_2_. As shown in Fig. 2*A*, *cmtR* expression was ∼3.8-fold up-regulated in the presence of 5 mm H_2_O_2_ compared with that in its absence. In contrast, the expression of the negative control gene *fbpB* remained unaffected under the same experimental conditions. This result indicates that H_2_O_2_ induces the expression of *cmtR* in the mycobacterial strain.

Next, we investigated the mechanism underlying the induction of *cmtR* expression by H_2_O_2_. CmtR contains a helix-turn-helix motif (Fig. 2*B*) and has been reported to specifically bind to the upstream fragment of the *cmtR* operon (36, 38). We confirmed the previous observation, as shown in the Fig. 2*C* When increasing quantities of CmtR (1.6–8.0 μm) were added to the reaction mixtures, the bands corresponding to CmtR and its promoter complex were observed to shift, and a corresponding increase was observed in the percentage of protein/DNA complexes (*lanes 2* and *3*). In contrast, CmtR could not bind with the *fbpBp* DNA substrate (Fig. S2*A*, *lanes 6*–*8*). Furthermore, ChIP assays were performed to investigate the specific binding of CmtR to its own promoter in *M. bovis* BCG. The *cmtR* promoter *cmtRp* could be specifically recovered using CmtR antibody (Fig. S2*B*). However, a negative control promoter (*fbpBp*) could not be recovered using CmtR antisera under similar conditions. The specificity of CmtR antibodies was confirmed using a *cmtR* deletion strain (Fig. S2*B*, *right panel*). These results indicate that *cmtRp* is the target promoter of CmtR in *M. bovis* BCG. We further assayed the effects of H_2_O_2_ on the DNA-binding ability of CmtR. As shown in Fig. 2*C*, a stepwise reduction in the concentration of the specific protein/DNA complex was observed upon the addition of 0.01–1 mm H_2_O_2_ into the reaction mixtures (*lanes 5*–*7*). Notably, this dissociation could be reversed with the addition of the reducing agent DTT (*lane 8*). No effect was observed when only 10 mm DTT was added into the reaction mixture (*lane 9*), which indicates that the ability of CmtR to bind to DNA was specifically inhibited in the presence of H_2_O_2_. This was confirmed in the quantitative ChIP assay. As shown in Fig. 2*D*, the ChIP and qPCR assays revealed that CmtR could precipitate ∼2.3-fold less promoter DNA of the *cmtR* operon in the presence of 5 mm H_2_O_2_ than in its absence. In contrast, no significant difference was observed when EthR was used as a control regulatory protein in the ChIP and qPCR assays under the same experimental conditions (Fig. S2*C*). Collectively, H_2_O_2_ inhibits the DNA-binding ability of CmtR both *in vitro* and in *M. bovis* BCG.

### Cys-24 residue of CmtR plays a unique role in H_2_O_2_ resistance of M. bovis

Cysteine residues have been shown to be responsible for redox-sensing in multiple transcriptional regulators (13). To identify the cysteine residue responsible for sensing oxidative stress in CmtR, we performed multiple sequence alignment of CmtR isolated from several mycobacterial species. As shown in Fig. S1*B*, six cysteine residues (Cys-4, Cys-24, Cys-35, Cys-57, Cys-61, and Cys-102) were conserved in certain important mycobacterial species, including *M. bovis* BCG and *M. tuberculosis* H37Ra and H37Rv (Fig. S1*B*). Site-directed mutations were further introduced at the sites encoding the cysteine residues in *cmtR*, and the DNA-binding activity of these mutant proteins was assayed in the electrophoretic mobility shift assay (EMSA). As shown in Fig. S3*A*, all the mutant proteins could retain their DNA-binding potential except for CmtR-C24C, the activity of which reduced marginally, and CmtR-C4S, which appeared to bind marginally more tightly than the WT protein. We next examined the effects of these mutations on the DNA-binding ability of CmtR under H_2_O_2_ stress. Notably, apart from that in CmtR-C24S, the mutations did not alter the sensitivity of CmtR to H_2_O_2_ (Fig. 2*E* and Fig. S3*B*). In contrast, treatment with H_2_O_2_ did not affect the DNA-binding activity of CmtR-Cys24S (Fig. 2*E*, *lanes 11–15*), which indicates that its potential to respond to the oxidative signal was not comparable with that of the WT protein. These results imply that the Cys-24 residue plays a unique role in responding to the H_2_O_2_ signal.

**Figure 3.**
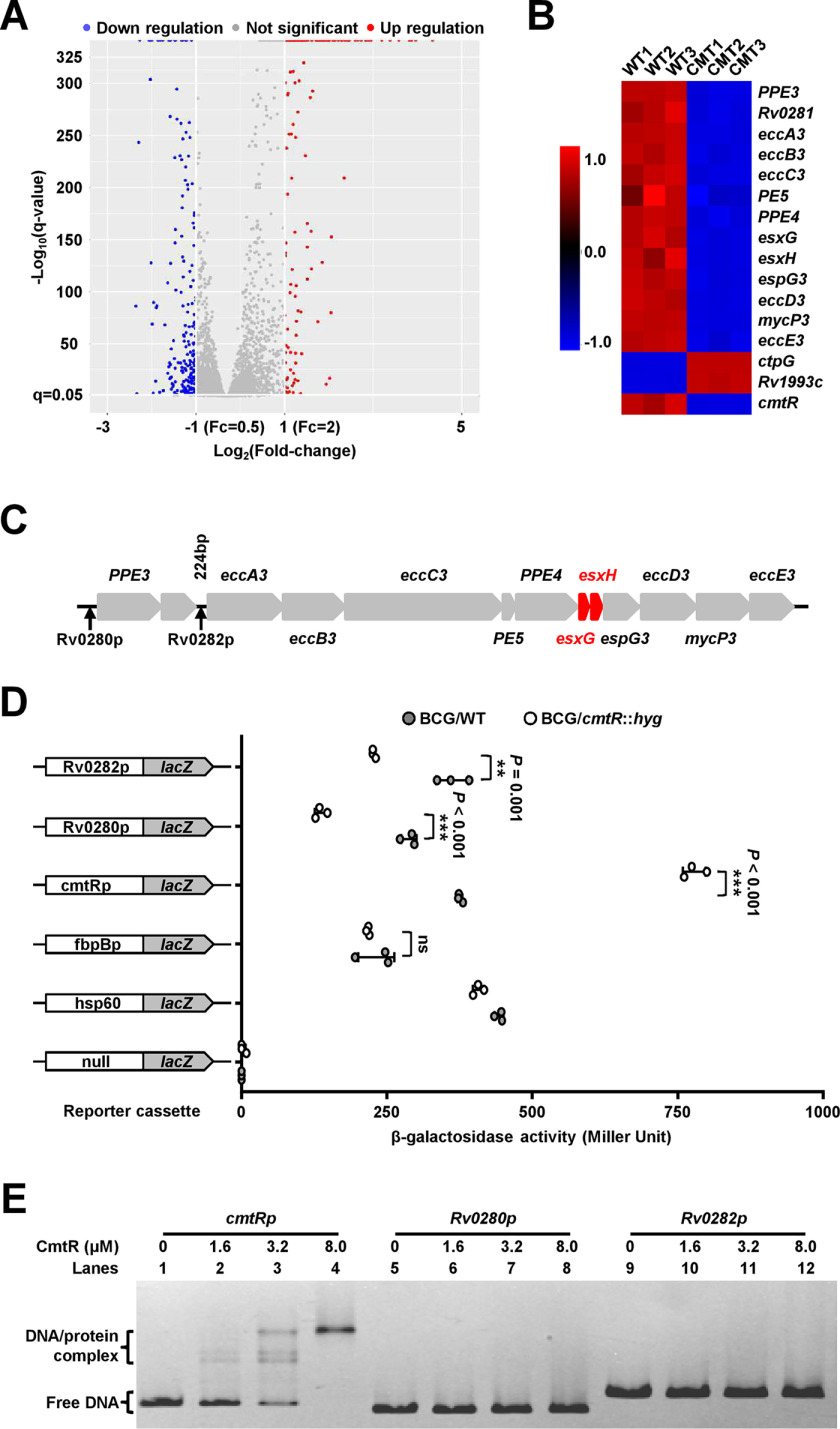
**Regulatory effect of CmtR on the expression of the *esx-3* operon.** *A*, volcano plot of the difference in gene expression between WT and *cmtR*-deleted *M. tuberculosis* H37Ra strains determined using RNA-Seq assays. The *x* axis and *y* axis indicate the log_2_(fold-change) values and the −log_10_(*q*-value) values, respectively, of all genes. The Cuffdiff program was executed to perform differential expression tests using the edgeR package. The differential expression of a transcript is reported as significant if the test indicates that the false discovery rate–adjusted *P*-value for multiple-testing represents statistically significant values (*q*-value < 0.05). The significantly up-regulated and down-regulated genes are indicated by *red* and *blue* spots, respectively. Genes that did not undergo significant changes in expression are indicated by *gray* spots. *B*, heat map of the CmtR-regulated differential expression profile for the *esx-3* operon and the *cmtR* operon. CMT1, CMT2, and CMT3 represent three biological replicates of differentially expressed genes in the *cmtR* knockout strain, respectively. WT1, WT2, and WT3 represent separately three biological replicates of the genes in the WT strain. *C*, the schematic of the *esx-3* operon and its regulatory region. Two noncoding regions (*rv0280*p and *rv0282*p), which were potentially recognized by CmtR, are indicated by *black arrows*. *D*, assays for the promoter activities of *rv0280*p and *rv0282*p in the presence or absence of *cmtR*. β-gal activity was evaluated in both WT and *cmtR*-deleted strains of *M. bovis* BCG. *Left column*: schematic representation of recombinant strain generation using reporter plasmids. Null promoter-*lacZ*, *hsp60*-*lacZ*, and *fbpB*p-*lacZ* were used as controls. *Error bars* represent the S.D. from three biological experiments. The *P*-values of the data were calculated by two-tailed Student's *t* test using GraphPad Prism 7. *Asterisks* represent significant difference (**, *P* < 0.01; ***, *P* < 0.001; *ns*, not significant, two-tailed Student's *t* test) between two groups. *E*, EMSA for studying the DNA-binding activity of CmtR to *cmtR*p promoter DNA (*lanes 1*–*4*), *rv0280*p (*lanes 5*–*8*), and *rv0282*p (*lanes 9*–*12*). The protein/DNA complexes and free DNA are indicated by *arrows* on the *left side* of the panels.

We further investigated the significance of the CmtR Cys24 residue in mycobacterial growth under H_2_O_2_ stress. As shown in Fig. S4*B*, the bacterial counts of both the *cmtR*-deleted strain and its *cmtR*-Cys24S complementation strain were significantly lower than that of the WT strain under 0.75 mm H_2_O_2_ stress for 4 and 6 days. In contrast, the WT *cmtR* gene could exactly complement the sensitive phenotype of the *cmtR*-deleted strain under H_2_O_2_ stress, and no growth difference was observed compared with the WT strain (Fig. S4*A*). These results indicate that the Cys-24 residue of CmtR plays a unique role in mycobacterial resistance to H_2_O_2_.

**Figure 4.**
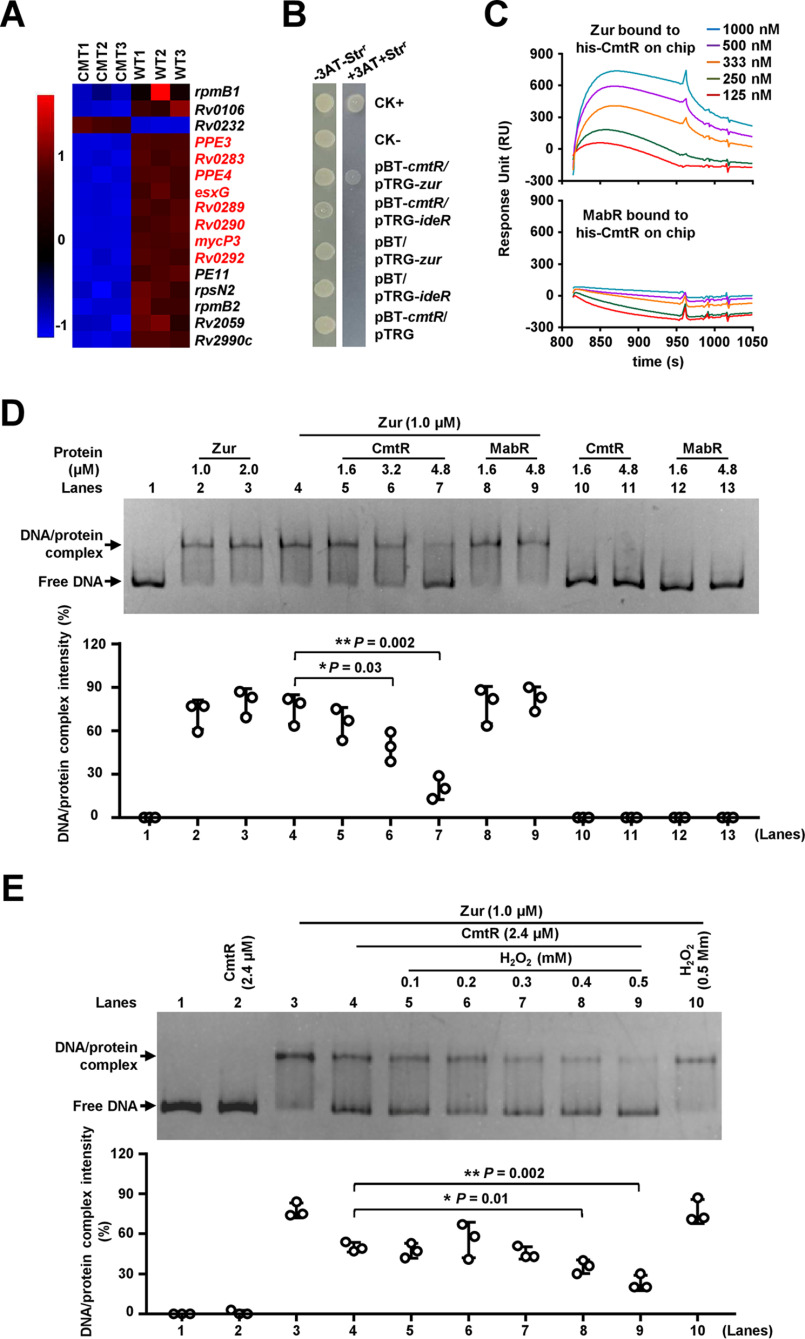
**Assays for studying the interaction between CmtR and Zur.** *A*, heat map of the CmtR-regulated differential expression profile of Zur-targeted genes. CMT1, CMT2, and CMT3 represent three biological replicates of the genes in the *cmtR* knockout strain. WT1, WT2, and WT3 represent three biological replicates of the genes in the WT strain. *B*, bacterial two-hybrid assays for the interaction between CmtR and Zur. *E. coli* reporter strains with various recombinant plasmids were spotted on the plate in the presence or absence of streptomycin (*str*) and 3-amino-1, 2, 4-triazole. *ideR* was used as a negative control. *C*, SPR assays for studying the specific interaction between CmtR and Zur. For studying the interaction between Zur and CmtR, His-tagged CmtR proteins were immobilized onto the NTA chips. Zur (*top panel*) protein and control MabR proteins (*bottom panel*) at different concentrations were passed over the chip. *D*, EMSA for studying the inhibitory effect of CmtR on the DNA-binding activity of Zur. The *rv0280*p DNA substrate was co-incubated with corresponding quantities of Zur (*lanes 2*–*4*), CmtR (*lanes 10* and *11*), or both (*lanes 5*–*7*). MabR was used as a negative control regulator. *E*, EMSA for studying the effect of H_2_O_2_ on the DNA-binding activity of Zur in the presence of CmtR. *Error bars* represent the S.D. from three independent experiments. The *P*-values of the data were calculated by unpaired two-tailed Student's *t* test using GraphPad Prism 7. *Asterisks* represent significant difference (*, *P* < 0.05; **, *P* < 0.01, two-tailed Student's *t* test) between two groups.

### CmtR triggers the expression of the esx-3 operon genes

To further study the signaling pathway through which CmtR regulates mycobacterial resistance to H_2_O_2_, we performed RNA-Seq and transcriptomic assays to compare the differential gene expression between the WT and *cmtR*-deleted strains. As shown in Fig. 3*A*, the expression levels of 365 genes were observed to be affected by *cmtR* knockout. Notably, the *esx-3* operon genes, which encode products essential for zinc and iron homeostasis, were integrally down-regulated in the *cmtR*-deleted strain compared with that in the WT strain (Fig. 3. *B* and *C*). In addition, the potential target regulatory genes of *cmtR* also include 12 inorganic ion transport- and metabolism-related genes, as shown in the evaluation of gene classification by the Cluster of Orthologous Groups analysis (Fig. S5). These data imply that CmtR may regulate metal ion homeostasis in mycobacterium.

**Figure 5.**
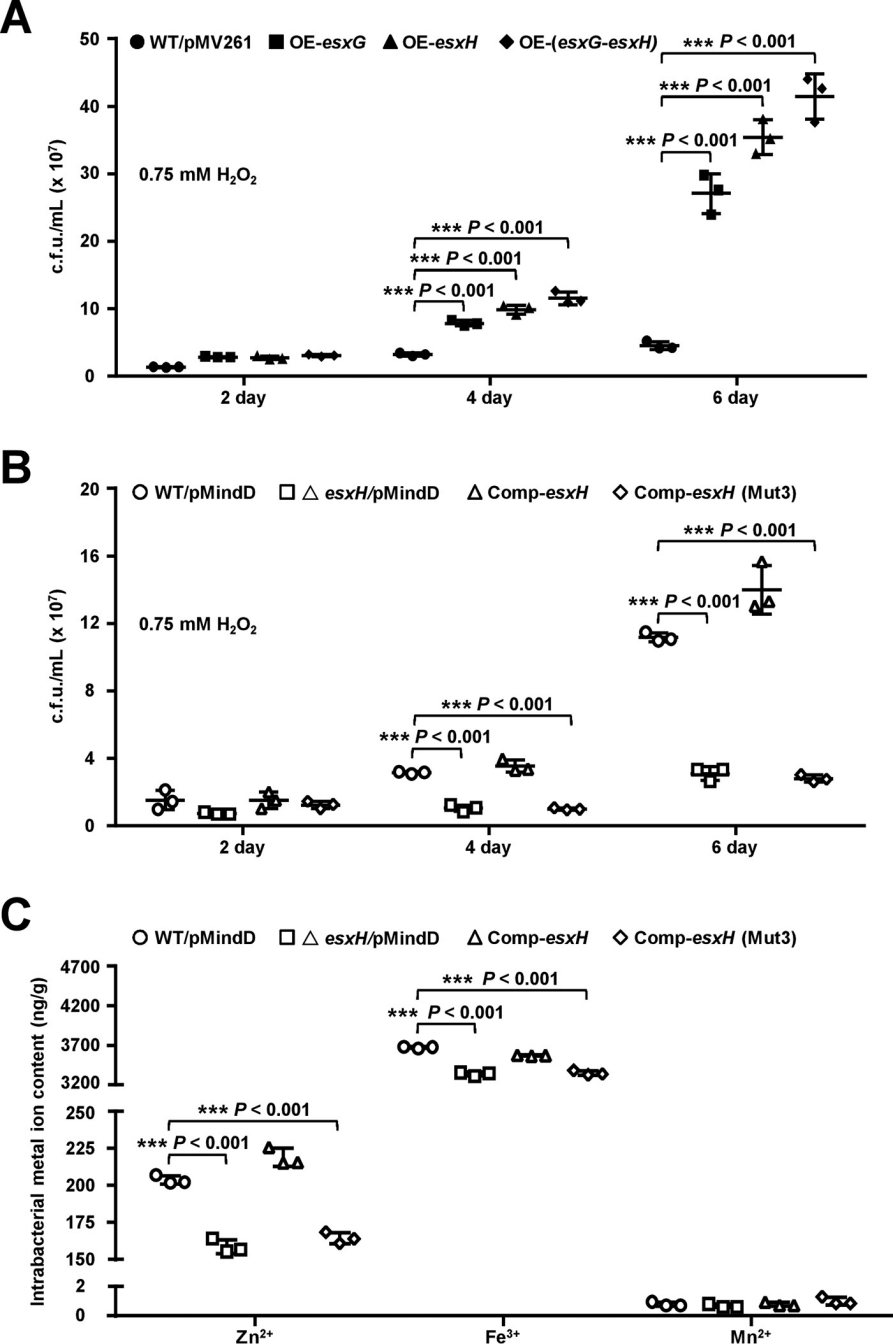
**Assays for studying the effects of EsxH on intracellular Zn^2+^ accumulation in bacteria and mycobacterial growth under H_2_O_2_ stress.** *A*, assays for studying the effects of *esxG* and *esxH* overexpression on the growth of *M. bovis* BCG strain under 0.75 mm H_2_O_2_ stress. WT/pMV261 represents the BCG/pMV261 strain; OE-*esxG*, OE-*esxH*, and OE-(*esxG*-*esxH*) represent the BCG*/*pMV261-*esxG*, BCG*/*pMV261-*esxH*, and BCG*/*pMV261-(*esxG-esxH*) strains, respectively. *B*, assays for studying the effects of *esxH* deletion on the growth of *M. bovis* BCG strain under 0.75 mm H_2_O_2_ stress. WT/pMindD represents the BCG/pMindD strain; △*esxH*/pMindD represents the BCG *esxH*::*hyg/*pMindD strain; Comp-*esxH* represents the BCG *esxH*::*hyg/*pMindD-*esxH* strain; Comp-*esxH*-Mut3 represents the BCG *esxH*::*hyg/*pMindD-*esxH* (Mut3) (Mut3: H14A, H70A, H76A) strain. *C*, assays for studying the effects of *esxH* on metal ion accumulation in bacterial cells under H_2_O_2_ stress. Mycobacterial strains were cultured till the *A*_600_ value reached 0.5 and were subsequently treated with 5 mm H_2_O_2_ for 24 h. The metal ion concentration within bacterial cells was measured by ICP-OES. *Error bars* represent the S.D. from three biological experiments. The *P*-values of the data were calculated by unpaired two-tailed Student's *t* test using GraphPad Prism 7. *Asterisks* represent significant difference (***, *P* < 0.001; two-tailed Student's *t* test) between two groups.

The positive regulation of the *esx-3* operon by CmtR in *M. bovis* BCG could be further confirmed by β-gal activity assays. As shown in Fig. 3*D*, *hsp60*p significantly promoted the expression of *lacZ* in different *M. bovis* BCG strains relative to the nonpromoter *lacZ* plasmid, which indicates that the reporting system functioned properly. *cmtR*p activated the expression of *lacZ* in the *cmtR*-deleted strain compared with that in the WT strain. In contrast, two *esx-3*p, *rv0280*p and *rv0282*p, significantly inhibited the expression of *lacZ* in the *cmtR*-deleted strain. However, no significant difference was observed in the expression of *lacZ* between the WT and *cmtR*-deleted strains when a negative control *fbpB*p was used as a promoter. These data indicate that although CmtR inhibits the expression of its own operon, it positively regulates the expression of the *esx-3* operon.

### The induction of the esx-3 operon by H_2_O_2_ depends on CmtR

A previous study revealed that the expression of the *esx-3* operon was induced by H_2_O_2_ in *M. tuberculosis* H37Rv (39). Here, we observed CmtR can act as a redox sensor and positively regulate the *esx-3* operon, which implies that H_2_O_2_ stimulates expression of the *esx-3* operon most likely through the CmtR regulator. To confirm this, we compared the expression of the *esx-3* operon genes in *M. bovis* BCG and *cmtR*-deleted strains in the presence or absence of oxidative stress. As shown in Fig. S6*A*, compared with that under no H_2_O_2_ stress, the expression of the *esx-3* operon genes in *M. bovis* BCG, including *Rv0281*, *esxG*, and *esxH*, was significantly up-regulated in the presence of 5 mm H_2_O_2_. In contrast, no significant difference was observed in the expression of these genes in the BCG/*cmtR*::*hyg* strain under similar conditions (Fig. S6*B*). However, *katG* expression was always induced in response to 5 mm H_2_O_2_ treatment both in the WT and *cmtR*-deleted strains (Fig. S6). These results indicate that the high expression of the *esx-3* operon (*esxG* and *esxH*) in response to H_2_O_2_ treatment depends on CmtR.

**Figure 6.**
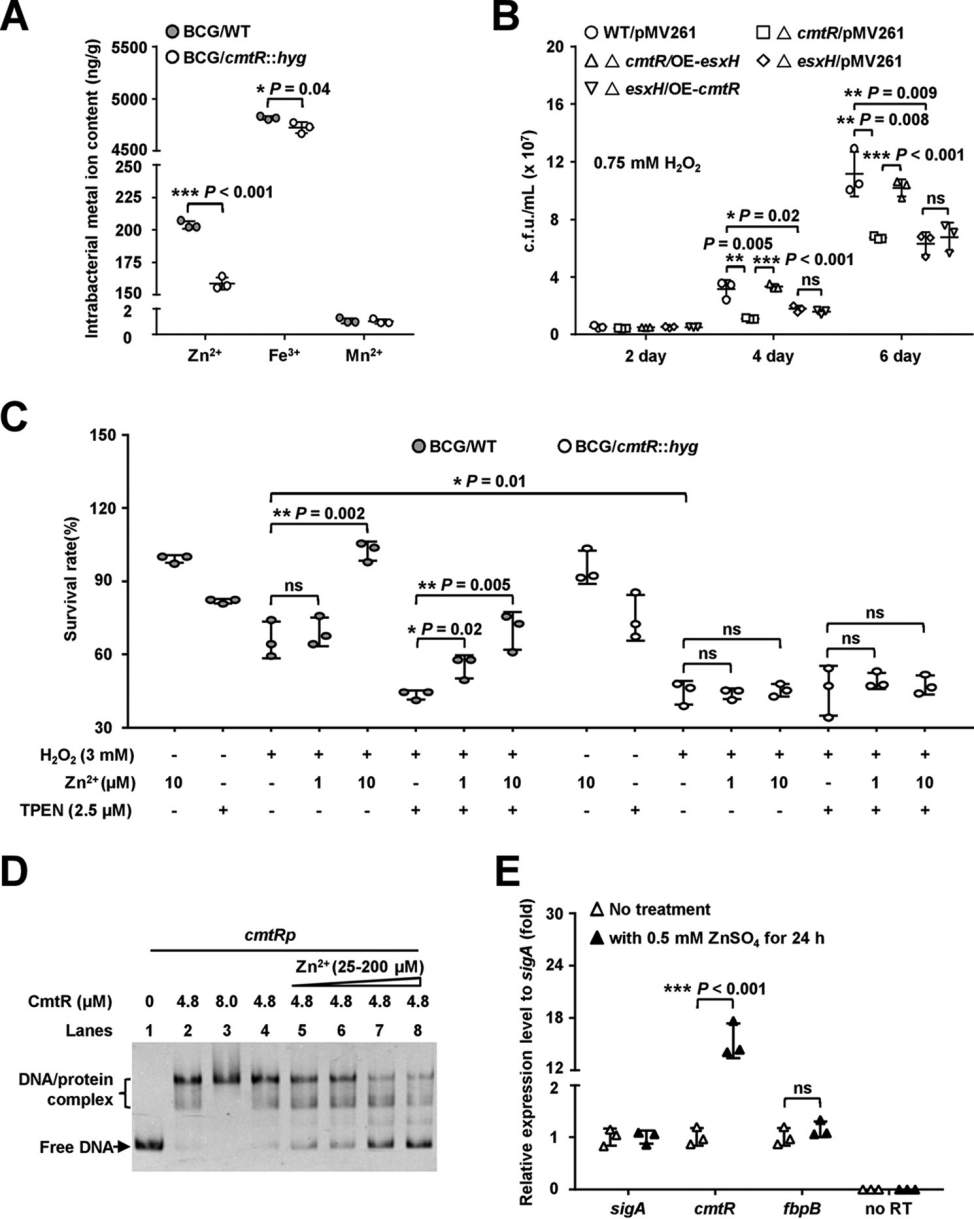
**Assays for studying the effect of CmtR on intracellular metal ion accumulation in bacteria and mycobacterial growth under oxidative stress.** *A*, ICP-OES assay for measuring the intracellular metal ion contents of WT and *cmtR*-deleted strains of *M. bovis* BCG under 5 mm H_2_O_2_ stress. *B*, assays for studying the effects of *esxH-*dependent regulation of *cmtR* on the growth of *M. bovis* BCG strain under 0.75 mm H_2_O_2_ stress. WT/pMV261 represents the BCG/pMV261 strain; △*cmtR/*pMV261 represents the BCG *cmtR*::*hyg/*pMV261 strain; △*cmtR/*OE-*esxH* represents the BCG *cmtR*::*hyg/*pMV261-*esxH* strain; △*esxH/*pMV261 represents the BCG *esxH*::*hyg/*pMV261 strain; △*esxH/*OE-*cmtR* represents the BCG *esxH*::*hyg/*pMV261-*cmtR* strain. *C*, assays for studying the essential role of CmtR in the enhancement of exogenous Zn^2+^ on antioxidant ability of *M. bovis* BCG strains. The c*mtR*-deleted and WT strains were cultured in Sauton's medium till the *A*_600_ value reached 0.5, and the culture was then supplemented with or without Zn^2+^, the Zn^2+^ chelator N, N, N′, N′-tetrakis (2-pyridylmethyl) ethylenediamine), and H_2_O_2_ for 24 h. *D*, EMSA for studying the effects of Zn^2+^ on the DNA-binding activity of CmtR. The *cmtRp* DNA substrate was co-incubated with CmtR in the absence (*lanes 1*–*4*) or presence of Zn^2+^ (25-200 μm) (*lanes 5*–*8*). *E*, qRT-PCR assays for studying the induction of *cmtR* expression in *M. bovis* BCG strain by Zn^2+^. *Error bars* represent the S.D. from three biological experiments. The *P*-values of the data were calculated by unpaired two-tailed Student's *t* test using GraphPad Prism 7. *Asterisks* represent significant difference (*, *P* < 0.05; **, *P* < 0.01; ***, *P* < 0.001; *ns*, not significant; two-tailed Student's *t* test) between two groups.

An EMSA was further performed to determine whether the purified CmtR protein can directly bind with the two *esx-3* operon promoter DNA substrates, *rv0280*p and *rv0282*p. As shown in Fig. 3*E*, as increasing levels of CmtR (1.6–8 μm) were added into the reaction mixtures, a stepwise increase was clearly observed in the levels of shifted DNA when CmtR was co-incubated with the *cmtR*p (*lanes 2*–*4*), whereas the same was not observed with *rv0280*p (*lanes 6*–*8*) and *rv0282*p (*lanes 10*–*12*), which indicates that CmtR alone could not directly bind with the *esx-3* operon promoter DNA. Collectively, these data indicate that CmtR could indirectly trigger the expression of the *esx-3* operon in response to H_2_O_2_ stress.

### CmtR physically interacts with Zur and neutralizes its inhibitory activity

Next, we focused on transcriptomic data to evaluate the mechanism underlying the activation of the *esx-3* operon by CmtR. Notably, except *Rv0232*, 16 genes inhibited by Zur (31) were significantly down-regulated in the *cmtR*-deleted strain (Fig. 4*A*). Of these, *rpmB1*(*Rv0106*), *rpmB2*(*Rv2059*), and the *esx-3* operon genes have been implicated in zinc homeostasis. Such overlap between target genes that are regulated by two transcriptional factors is indicative of direct interaction between CmtR and Zur. To confirm this, we first studied the interaction in bacterial two-hybrid assays. IdeR has been reported to directly regulate the expression of the *esx-3* operon (32), and was used as a negative control regulator. As shown in Fig. 4*B*, the co-transformant containing *cmtR*/*zur* was cultured successfully in the screening medium, which indicates that CmtR directly interacts with Zur. In contrast, the co-transformant containing *cmtR*/*ideR* did not grow under similar screening conditions. The controls containing *cmtR*, *zur*, or *ideR* alone did not grow under the same conditions. These results indicate that CmtR specifically interacts with Zur. Using purified transcriptional factor proteins for surface plasmon resonance (SPR) analysis, we confirmed the physical interaction between these two regulators. As shown in Fig. 4*C*, the corresponding response increased at increasing concentrations of Zur (125–1000 nm) over the His-tagged CmtR-immobilized nitrilotriacetic acid (NTA) chip. In contrast, no clear interaction was observed between CmtR and MabR under similar conditions. Therefore, Zur and CmtR interact physically.

The direct interaction suggests that CmtR can affect the regulation of *esx-3* operon expression by Zur. To confirm this, we first studied the effect of CmtR on the DNA-binding ability of Zur with the *esx-3* operon promoter (*rv0280*p) by EMSA. As shown in Fig. 4*D*, with the addition of increasing levels (1.6–4.8 μm) of CmtR to the reaction mixture, a stepwise reduction was observed in the levels of shifted bonds corresponding to the specific Zur and *rv0280*p DNA complex (*lanes 5*–*7*). In contrast, no obvious effect was observed for MabR, an unrelated protein, under the same experimental conditions (*lanes 8* and *9*), which indicates that CmtR specifically inhibits the DNA-binding ability of Zur.

We further studied the integrated effect of H_2_O_2_ and CmtR on the DNA-binding activity of Zur using EMSA. As shown in Fig. 4*E*, at 1 μm, Zur could bind satisfactorily with *rv0280*p (*lane 3*). No bond shift was observed, as only 3.2 μm CmtR was added into the DNA-binding reaction mixture (*lane 2*). Upon the addition of 2.4 μm CmtR into the reaction mixture, a clear decrease was observed in the levels of shifted bonds (*lanes 4*). Notably, upon the addition of increasing concentrations of H_2_O_2_ (0.1–0.5 mm) to the reaction mixtures, the DNA substrates shifted by Zur were released continuously (*lanes 5*–*9*). These results indicate that H_2_O_2_ further enhances the CmtR-mediated inhibition of the DNA-binding activity of Zur.

Collectively, CmtR physically interacts with Zur and neutralizes its inhibitory activity. H_2_O_2_ can further enhance the CmtR-mediated inhibition of the DNA-binding activity of Zur.

### Regulation of bacterial growth by CmtR depends on esxH expression

CmtR positively regulates the expression of the *esx-3* operon genes, including *esxG* and *esxH*, by inhibiting the DNA-binding ability of Zur, and enhances mycobacterial H_2_O_2_-resistance, which implies that EsxG or EsxH contributes to mycobacterial resistance to oxidative stress. To confirm this, we studied the effects of *esxG*, *esxH*, and *esxG-esxH* overexpression on mycobacterial growth under H_2_O_2_ stress_._ As shown in Fig. 5*A*, when the plasmid pMV261 was used to overexpress *esxG*, *esxH*, or *esxG*-*esxH* in *M. bovis* BCG, the recombinant mycobacterial strains exhibited better growth than the WT strain when treated with 0.75 mm H_2_O_2_ for 4 and 6 days. No obvious growth difference was observed among these mycobacterial strains in the absence of H_2_O_2_ stress at the specified time points (Fig. S7*A*). These results indicate that EsxG or EsxH increases mycobacterial resistance to H_2_O_2_. We further constructed an *esxH*-deleted strain and constructed both WT *esxH* and its mutant gene *esxH(Mut3)*, in which three zinc-binding amino acids (H14A, H70A, and H76A) were mutated, to form complementation *M. bovis* BCG strains. As shown in Fig. 5*B*, the bacterial counts of the *esxH*-deleted strain and the *esxH3(Mut3)* complementation strain were found to be significantly lower than those of the WT strains when treated with 0.75 mm H_2_O_2_ for 4 and 6 days. In contrast, no growth difference was observed among the strains in the absence of H_2_O_2_ at the specified time points (Fig. S7*B*). These results further demonstrate that EsxH contributes to the ability of mycobacteria to survive under oxidative stress.

**Figure 7.**
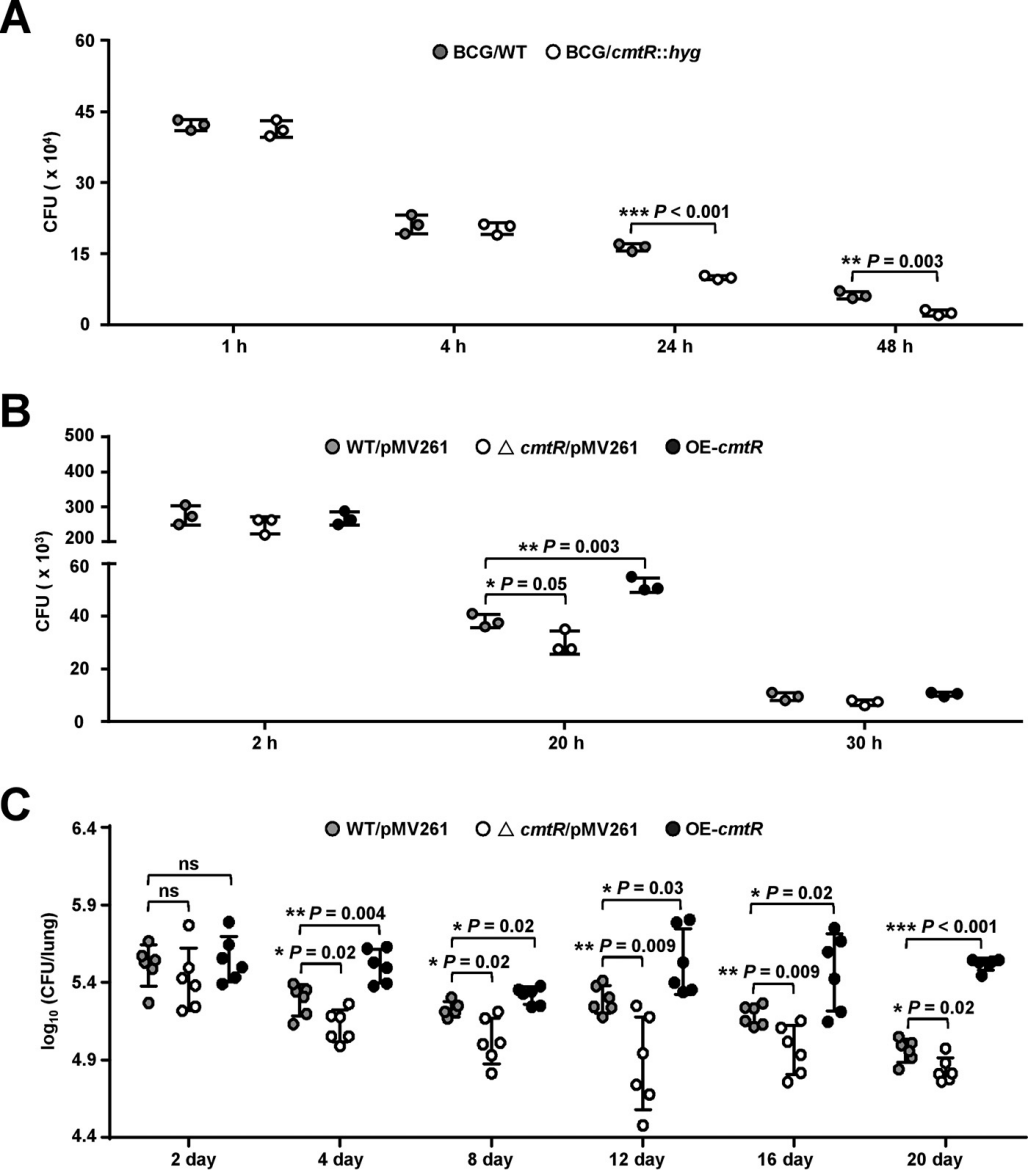
**Assays for studying the effect of CmtR on intracellular survival of mycobacteria in macrophages and in mice.** Cells were infected with mycobacterial strains at a multiplicity of infection of 10 and washed three times at 4 h post infection to remove extracellular bacteria. Thereafter, the cells were re-incubated in a medium supplemented with penicillin/streptomycin and lysed using 0.025% SDS at the indicated post-infection time points. Serial dilutions of the supernatant were then plated on 7H10 agar supplemented with 10% oleic acid–albumin–dextrose–catalase, and the number of cfu (*CFU*) was counted 15–21 days later. *A*, RAW264.7 cells were infected with BCG/WT and BCG/*cmtR*::*hyg* strains separately. *B*, BMDMs were infected with WT/pMV261 (BCG/pMV261), △*cmtR*/pMV261 (BCG *cmtR*::*hyg/*pMV261), and OE-*cmtR* (BCG/pMV261-*cmtR*) strains, respectively. *C*, female SPF C57BL/6 mice (*n* = 6 mice per group) were infected intratracheally with 1 × 10^6^ of WT/pMV261, △*cmtR*/pMV261, and OE-*cmtR* strains for 0–20 days, and the bacterial loads in lung tissue homogenates of mice were determined. *Error bars* represent the S.D. from three biological experiments. The *P*-values of the data were calculated by unpaired two-tailed Student's *t* test using GraphPad Prism 7. *Asterisks* represent significant difference (*, *P* < 0.05; **, *P* < 0.01; ***, *P* < 0.001; *ns*, not significant; two-tailed Student's *t* test) between two groups.

The EsxG-EsxH complex had been observed to possess a specific Zn^2+^-binding site formed from a cluster of histidine residues in EsxH (34), which implies that their expression levels have a potentially important effect on bacterial zinc homeostasis, and the regulation of mycobacterial growth potential under antioxidant stress by CmtR could be related to zinc homeostasis. To confirm this, we determined the zinc concentrations in several recombinant strains using inductively coupled plasma optical emission spectrometry (ICP-OES). As shown in Fig. 5*C*, consistent with the findings of the previous study, the intracellular Fe^3+^ contents in the *esxH*-deleted strain and the *esxH(Mut3)* complementation strain were significantly lower than that in the WT strain under 5 mm H_2_O_2_ stress. Notably, a similar result was obtained for the intracellular Zn^2+^ content (Fig. 5*C*). In contrast, no difference was observed in the intracellular Mn^2+^ content of these strains under the same experimental conditions. These results indicate that EsxH contributes to the antioxidant potential of mycobacteria and affects zinc homeostasis in mycobacterium.

### CmtR regulates EsxH-dependent Zn^2+^ accumulation in M. bovis BCG

CmtR triggers the expression of the *esx-3* operon genes, including *esxH*, the product of which affects zinc homeostasis in *M. bovis*, which implies that CmtR may regulate the intracellular Zn^2+^ content under oxidative stress. To confirm this, we assayed the Zn^2+^ content in the WT and *cmtR*-deleted strains. As shown in Fig. 6*A*, the levels of both intracellular Zn^2+^ and Fe^3+^ in the *cmtR*-deleted *M. bovis* strain were significantly lower than those in the WT strain under 5 mm H_2_O_2_ stress. In contrast, the Mn^2+^ content remained unaffected in the *cmtR*-deleted strain under the same experimental conditions. These data suggest that CmtR is essential for the intracellular accumulation of Zn^2+^ and Fe^3+^ under H_2_O_2_ stress.

Further, we confirmed that the effect of CmtR on mycobacterial H_2_O_2_ resistance depends on the expression of *esxH* and the intracellular accumulation of Zn^2+^. We first determined if CmtR-mediated regulation of mycobacterial H_2_O_2_ resistance depends on the expression of EsxH. As shown in Fig. 6*B*, compared with the WT strain, the growth of the *cmtR*-deleted or *esxH*-deleted strains was significantly inhibited under 0.75 mm H_2_O_2_ stress for 4 and 6 days. When *esxH* was overexpressed in the *cmtR*-deleted strain, the recombinant strain exhibited better growth than the *cmtR*-deleted strain; however, when *cmtR* was overexpressed in the *esxH*-deleted strain, no obvious growth difference was observed under similar stress conditions. In contrast, no growth difference was observed among these strains in the absence of H_2_O_2_ stress (Fig. S7*C*). In addition, the survival rates of the *cmtR*-deleted or *esxH*-deleted strains were significantly lower than that of the WT strain, as shown in Fig. S8. When *esxH* was overexpressed in the *cmtR*-deleted strain, the survival rate of the recombinant strain was higher than that of the *cmtR*-deleted strain; however, no obvious difference was observed when *cmtR* was overexpressed in the *esxH*-deleted strain. These results indicate that the effect of CmtR on mycobacterial H_2_O_2_ resistance depends on EsxH.

**Figure 8.**
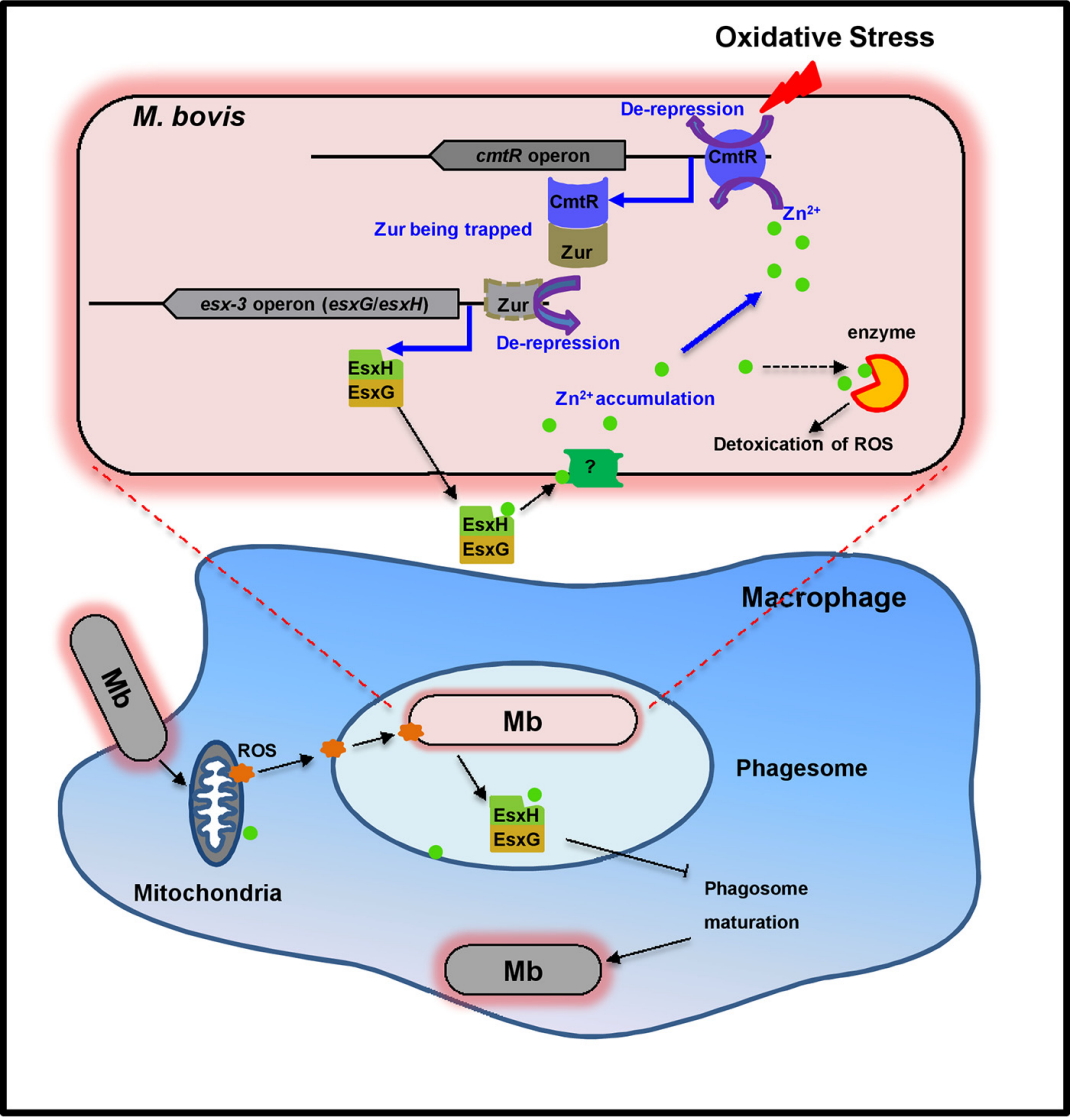
**A model depicting that the CmtR-ESX3-Zn^2+^ signaling pathway triggered by oxidative stress contributes to the survival of *M. bovis* in the host.** Upon sensing an oxidative stress signal, CmtR expression is induced significantly, and it targets the negative regulator Zur to de-repress its inhibition on the expression of *esxG* and *esxH*, which triggers the accumulation of Zn^2+^ in the cytoplasm of *M. bovis*. Zn^2+^ subsequently activates antioxidant enzymes for combating oxidative stress in the host cell. Conversely, Zn^2+^ can de-repress the self-inhibition of *cmtR*, and further trigger the antioxidant regulatory signaling pathway.

Next, we evaluated whether the intracellular accumulation of Zn^2+^ contributes to bacterial survival under H_2_O_2_ stress. As shown in Fig. 6*C*, the WT strain exhibited better growth than the *cmtR*-deleted strain in the presence of 3 mm H_2_O_2_. Under 3 mm H_2_O_2_ stress, the addition of exogenous Zn^2+^ led to the rescue of the growth of the WT strain, whereas that of the *cmtR*-deleted strain was not rescued. Furthermore, the inhibitory effects of TPEN and H_2_O_2_ could be partly neutralized by increasing the concentration of exogenous Zn^2+^ in a dose-dependent manner in the WT strain culture, whereas the same could not be achieved in the *cmtR*-deleted strain culture. These data indicate that the intracellular accumulation of Zn^2+^ contributes to the potential of growth under H_2_O_2_ stress in mycobacteria.

### Zn^2+^ inhibits the DNA-binding activity of CmtR both in vitro and in M. bovis

It has been shown previously that CmtR binds Zn^2+^ *in vitro* (37), which implies that Zn^2+^ may affect the DNA-binding activity of CmtR. An EMSA was conducted to validate this possibility. As shown in Fig. 6*D*, CmtR (4.8–8 μm) could bind satisfactorily with the *cmtR*p DNA substrate (*lanes 2* and *3*). When increasing quantities of Zn^2+^ (25–200 μm) were added to the reaction mixtures (*lanes 5*–*8*), the levels of shifted DNA substrates decreased correspondingly, which indicates that Zn^2+^ inhibited the DNA-binding activity of CmtR. The findings from the qRT-PCR assay further confirmed the effect of Zn^2+^ on the expression of *cmtR* in *M. bovis* BCG strain. As shown in Fig. 6*E*, *cmtR* expression was up-regulated by ∼15-fold in the presence of 0.5 mm Zn^2+^ compared with that under no treatment. In contrast, the expression of the control gene *fbpB* remained unaffected under the same experimental conditions. These results indicate that Zn^2+^ inhibits the DNA-binding activity of CmtR and induces the expression of *cmtR* in mycobacterial strains.

### CmtR enhances M. bovis survival within the host

CmtR enhances the ability of *M. bovis* to proliferate under oxidative stress and promotes the expression of *esxH*, which implies that this regulator could contribute to bacterial survival in the host. To confirm this, we utilized two cell models to evaluate the effects of CmtR on the survival of *M. bovis*. First, RAW264.7 macrophages were infected with WT and *cmtR*-deleted strains. The results revealed that both strains invaded macrophages comparably at 1 h post infection (hpi). However, the intracellular survival efficiency of the *cmtR*-deleted strain in macrophages was significantly lower than that of the WT strain at 24 and 48 hpi (Fig. 7*A*). Similarly, the survival efficiency of the *cmtR*-deleted strain was significantly lower than that of the WT strain in bone marrow–derived macrophages (BMDMs) at 20 and 30 hpi, whereas the survival efficiency of the *cmtR*-overexpressing strain was observably higher than that of the WT strain (Fig. 7*B*). These results strongly suggest that CmtR enhances mycobacterial survival in macrophages.

Next, we determined the bacterial loads in the lungs of C57BL/6 mice infected with the WT, *cmt*R-deleted, and *cmtR*-overexpressing *M. bovis* strains. As shown in Fig. 7*C*, the bacterial load in the lung tissues of mice infected with all three strains were similar at 2 days post infection (dpi); however, the bacterial load in the lungs of mice infected with the *cmtR*-overexpressing strain increased significantly compared with that of mice infected with the WT strain from 4 to 20 dpi. In contrast, compared with the mice infected with the WT strain, those infected with the *cmtR-*deleted strain exhibited an obvious decrease in bacterial load since 4 dpi, which indicates that CmtR enhances the *M. bovis* load in the lungs of mice. Collectively, our data suggest that CmtR enhances mycobacterial survival in macrophages *in vitro* as well as in mice.

## Discussion

Mtb is considered one of the most persistent intracellular pathogens and has developed unique mechanisms to adapt to host environments during infection. However, upon sensing oxidative stress, the molecular mechanism by which mycobacteria adaptively promote antioxidant regulation, and the associated signaling pathways, remains largely unclear. In this study, using *M. bovis* BCG as a model, we first characterized CmtR as a novel redox sensor containing an essential cysteine residue for sensing oxidative stress signals. We further observed that CmtR can physically interact with Zur and is associated with the expression regulation of the *esx-3* operon and maintenance of Zn^2+^ homeostasis in bacterial cells. Lastly, we provided evidence to demonstrate that CmtR affects mycobacterial interaction with host cells and contributes to bacterial survival under stress and during infection. Our findings revealed the existence of a novel antioxidant defense pathway and regulatory mechanism that mycobacteria respond to and adapt to in the host environment.

To date, only a number of redox-sensing regulators have been characterized in mycobacterial species, including MosR (18) and OxyS (19). Notably, these regulators function as redox-dependent transcription factors that can directly recognize the upstream regulatory sequence of the redox gene cluster. For example, MosR senses and responds to oxidative stress and regulates the expression of a putative exported oxidoreductase (18), whereas OxyS regulates the expression of *katG* (19). This represents the model for the regulation of antioxidant defense in bacteria by a redox sensor. In the present study, we successfully characterized CmtR as a novel redox sensor, which contains an essential cysteine residue for sensing redox signals. Moreover, we found that CmtR does not directly bind with the upstream regulatory sequence of the *esx-3* operon; instead, it physically interacts with Zur, a zinc uptake repressor of the operon (31). This eventually leads to the de-repression of Zur regulation and up-regulates the expression of the *esx-3* operon, which enhances Zn^2+^ accumulation in bacterial cells and triggers ROS detoxication. Therefore, in contrast to the mechanism followed by redox sensors reported earlier, the regulation of antioxidant defense by CmtR represents a novel model for antioxidant defense in mycobacteria.

Metal ions are strongly associated with bacterial survival under oxidative stress. Fe^2+^ ions are widely associated with such conditions, and they frequently cause oxidative stress via the Fenton reaction in the presence of oxygen. Zn^2+^ is an essential trace element localized to the active center or in a structurally important site in several bacterial proteins (23, 40). In particular, Zn^2+^ is also a cofactor for more than 300 enzymes, including several oxidoreductases, such as superoxide dismutase and alcohol dehydrogenase (40). Therefore, Zn^2+^ homeostasis is related to bacterial adaptation to oxidative stress. Meanwhile, certain intracellular pathogens must adapt to host-imposed zinc toxicity (41, 42). Notably, in *M. tuberculosis*, ESX-3, one of the type-VII secretion systems, was recently found to have a zinc transportation function. Interestingly, the expression of the *esx-3* operon is regulated by Zur to maintain zinc homeostasis in *M. tuberculosis* (31, 33). In the present study, we found that CmtR can physically interact with Zur and inhibit the Zur-mediated repression of *esx-3* operon. Deletion of *cmtR* significantly inhibited the *esx-3* operon expression and bacterial growth under oxidative stress. This links CmtR to the regulation of the operon and bacterial survival under both oxidative stress and during infection. Consistently, we provided data to confirm that the overexpression of the special transport system product, EsxH, could improve Zn^2+^ concentration in bacterial cells and the corresponding potential for bacterial growth under oxidative stress. This is a novel regulatory pathway for mycobacterial adaptation to a stressful environment.

An interesting finding from the present study is that by influencing the expression of a type-VII secretion system, the function of CmtR, a redox-sensing regulator, could be linked to the interaction between mycobacteria and the host. The genome of *M. tuberculosis* encodes five ESX type-VII secretion systems, which can export a variety of proteins linked to tuberculosis pathogenesis (25, 26). ESX-3 is conserved in several mycobacterial species and can function as zinc transporter. Meanwhile, one of the ESX components, EsxH, can target the host endosomal sorting complex to impair phagosome maturation and the recognition of Mtb-infected cells (27, 28). In addition, the expression and secretion of the EsxG-EsxH heterodimer complex have been found to significantly affect *M. tuberculosis*–macrophage interaction and bacterial survival in host cells (29, 30). Consistently, in the present study, we observed that CmtR promotes mycobacterial virulence. Mice infected with the *cmtR*-overexpressing *M. bovis* BCG strain exhibited a higher bacterial load in the lungs than those infected with the WT strain. These are consistent with the finding that CmtR positively affected the expression of the *esx-3* operon, as well as with findings from previous reports.

In summary, we characterized CmtR as a novel redox sensor in *M. bovis* BCG and showed that the regulation antioxidant defense by CmtR is a part of a novel signaling pathway. Our data support a model in which the expression of the redox sensor CmtR is induced in response to oxidative stress, following which CmtR targets the repressor Zur and de-represses the *esx-3* operon (Fig. 8). On one hand, EsxH and EsxG transport Zn^2+^ into bacterial cells to promote ROS detoxication. On the other hand, both EsxH and EsxG impair phagosome maturation and the recognition of Mtb-infected cells. Both pathways aid mycobacterial survival during infection. Our findings reveal a novel signaling pathway and the molecular mechanism underlying antioxidant defense in *M. bovis* and provide insights into stress-induced mycobacterial adaptation to the host environment.

## Experimental procedures

### Plasmids, enzymes, and reagents

The pET28a and pET28a-SUMO plasmids obtained from Novagen were used for overexpressing the mycobacterial protein in *E. coli* BL21 (DE3) strain (Novagen, Darmstadt, Germany). The pBT and pTRG vectors and the *E. coli* XR strains used for the bacterial two-hybrid assay were purchased from Stratagene (La Jolla, CA, USA). The primers for PCR were synthesized by Tsingke (China); these have been listed in Table S1. DNA polymerase, restriction enzymes, T4 ligase, dNTPs, and the antibiotics used were obtained from TaKaRa Biotech (Shiga, Japan). Ni^2+^-NTA–agarose was purchased from Qiagen (Hilden, Germany). The 7H9/7H10 medium and oleic acid–albumin–dextrose–catalase enrichment, which were used for mycobacterial growth, were purchased from BD Biosciences. Antisera were obtained from the Wuhan Animal Center of the Chinese Academy of Sciences (Wuhan, China).

### Expression and purification of recombinant proteins

Genes were amplified using PCR with specific primer pairs (5′-CCACGAATTCGTATGCTGACGTGTGAGATGCG-3′ and 5′-CCGATCTAGATCTCAGCTACCTGTCATCTCGA-3′ for *cmtR*; 5′-AAAAATGGATCCATGAGTGCAGCCGGTGTC-3′ and 5′-CCAAAAGCTTTTAGCTC CGGCAGTCTGA-3′ for *zur*; 5′-ATTAGGATCCGTGAACGACAATCAGTTGGC-3′ and 5′-ATTAAAGCTTTCAGTGCGGCGTCGGATAGT-3′ for *mabR*). Several mutant genes were obtained through site-directed mutagenesis by overlapping extension PCR. The amplified DNA fragments were digested using the corresponding restriction endonucleases and cloned into the pET-28a or pET28a-SUMO expression vectors to produce recombinant plasmids (Table S2). The expression strains of *E. coli* BL21 (DE3) containing the recombinant plasmids were cultured, and the recombinant proteins were purified, as described in a previous study (43). The SUMO fusion protein was cleaved using the SUMO protease ULP1 to remove the tag (44). The eluate was dialyzed using dialysis buffer (20 mm Tris-HCl, pH 7.5, 100 mm NaCl, 1 mm l-Arg, 10% glycerin) for 4 h at 4°C and stored at −80°C. Protein concentration was determined using the Coomassie Brilliant Blue assay.

### EMSA

The DNA substrates for EMSA were amplified from *M. tuberculosis* H37Rv genomic DNA by PCR. The sequences of oligonucleotides fragments are listed in Table S1. The DNA-binding ability of proteins was evaluated using modified EMSA, as described previously (43). Briefly, the reaction mixtures (20 μl) for measuring mobility shift contained DNA fragments, Zur/CmtR/MabR at various concentrations, and buffer (20 mm Tris-HCl, pH 7.5, 100 mm NaCl, 1 mm l-Arg, 10% glycerin) in the presence or absence of H_2_O_2_ at different concentrations. First, the proteins and H_2_O_2_ (at the indicated concentration) were co-incubated for 10 min on ice in dark. Next, the DNA substrates were added to the reaction mixtures and incubated for 20 min on ice. Lastly, the mixtures were electrophoresed in a 5% native polyacrylamide gel containing 1× Tris-glycine buffer at 150 V. Images were recorded using an FLA-5100 Fluorescent Image Analyzer (FUJIFILM, Japan).

### Construction of recombinant mycobacterial strains

*esxG, esxH*, and *esxH* (H14A, H70A, H76A) genes were amplified by PCR using specific primer pairs (Table S1), and the amplicons were digested by the corresponding restriction endonucleases. The digested *esxG*, *esxH*, *esxG-esxHI*, and *cmtR* fragments, along with the corresponding mutant genes (mentioned above), were separately cloned into pMV261 overexpression vectors (45) or a pMindD vector (46) and transformed into *M. bovis* BCG WT and knockout strains to generate overexpression and complementary strains, respectively, in which the expression of the target genes was regulated using anhydrotetracycline hydrochloride. *cmtR* or *esxH* knockout was performed in *M. bovis* BCG or *M. tuberculosis* H37Ra strains, as described previously (47).

### ChIP assay

The ChIP assay was performed as described previously with certain modifications (43). WT and *cmtR*-deleted *M. bovis* BCG strains were cultured until the *A*_600_ value reached 0.6 in 100 ml of 7H9 medium, after which the WT strains were separately supplemented with 0 or 5 mm H_2_O_2_ and cultured for 24 h. The bacterial cells were fixed with 1% formaldehyde, and the reactions were terminated using 0.125 M glycine. Next, the crosslinked cells were harvested and resuspended in 1 ml of TBST (TBS, 0.2% Triton X-100, 0.05% Tween 20). The sample was sonicated on ice, and the average DNA fragment size was determined to be 0.5–1.0 kb. The supernatant was collected from centrifuged samples. The special antibodies or pre-immune sera were added into 100 μl of the sample extracts under rotation for 3 h at 4°C. The complexes were immunoprecipitated by treating with 50 μl of 50% protein A-agarose for 1 h at 4°C. The immune complexes were recovered by centrifugation and resuspended in 50 μl TE (20 mm Tris-HCl, pH 7.8, 10 mm EDTA, 0.5% SDS). Next, the crosslinking was reversed by treatment for 6 h at 65°C. The DNA samples from the input and ChIP assay were purified and analyzed by PCR. The primer sequences are listed in Table S1. The protocol included one denaturation step of 5 min at 95°C, followed by 25 cycles of 20 s at 95°C, 20 s at 60°C, and 30 s at 72°C. The PCR products were separated by electrophoresis in 1.5% agarose gel containing 1 × Tris acetate-EDTA buffer at 100 V for 30 min.

### Quantitative real-time PCR assays

mRNA extraction from the WT and *cmtR*-deleted *M. bovis* BCG strains and real-time PCR analysis were performed subsequently, as described earlier (19).

### Quantitative ChIP assay

Quantitative ChIP assay is a quantitative PCR (qPCR) approach used for ChIP. The purified DNA samples of the input and ChIP assay were analyzed by qPCR (19).

### Transcriptomic analysis

*M. tuberculosis* H37Ra strains (H37Ra/WT, H37Ra/*cmtR*::*hyg*) were cultured in 7H9 medium and subjected to shaking at 160 rpm at 37°C. The cells were cultured until the mid-logarithmic phase and harvested from each sample (each strain in three biological replicates). Subsequent transcriptomic analysis was performed, as described previously (47). In brief, total RNA was isolated using the RNeasy mini kit (Qiagen, Germany). Strand-specific libraries were prepared using the TruSeq^®^ Stranded Total RNA Sample Preparation Kit (Illumina, USA) according to the manufacturer's instructions. Library construction and sequencing were performed at Shanghai Biotechnology Corporation.

The volcano plot diagrams were constructed using the −log_10_(q-value) and log_2_(fold-change) values of the genes between the WT and *cmtR*-deleted *M. tuberculosis* H37Ra strains using the ggplot2 package. Briefly, the cuffdiff program (48) was performed to conduct differential expression tests between the WT and *cmtR* knockout samples using the edgeR package (49). A transcript will be reported as differential expression significant if the test gives that the false discovery rate–adjusted *P*-value after Benjamini-Hochberg (50) correction for multiple-testing represents statistical significant (*q*-value < 0.05) (48, 51). The changes in gene expression are indicated on the *x* axis, and the *q*-values are indicated on the *y* axis. The *red* and *blue* spots represent the up-regulated and down-regulated genes, respectively, whereas the *gray* spots represent genes with insignificant changes in expression (Fig. 3A).

The heat map was constructed using the HemI (Heatmap Illustrator, version 1.0) software. Briefly, the fragments per kilobase of exon per million fragments mapped values of target genes in each sample were normalized and imported to the HemI software to construct the heat map diagram. *Red* represents high expression, whereas *blue* represents low expression of the target genes in different samples. The color scale beside the heat map indicates the color threshold.

### Assay for β-gal activity

An assay for measuring β-gal activity was performed using the WT and *cmtR*-deleted *M. bovis* BCG strains by constructing operon-*lacZ* fusions based on the expression vector pMV261 (52). The target and control promoters were amplified by PCR using the respective primers, which are listed in Table S1, after which the amplicons were digested using the corresponding restriction endonucleases and cloned into the pMV261 backbone. The reporter gene *lacZ* (Table S1) was cloned downstream of the promoters. The plasmids were separately transformed into the *cmtR*-deleted and WT *M. bovis* BCG strains to obtain the corresponding recombinant reporter strains. The recombinant strains were cultured until the mid-logarithmic phase in 7H9 medium at 37°C. The bacterial cells were harvested and washed using PBS. The levels of galactosidase were measured as described previously (47).

### Bacterial two-hybrid assay

The BacterioMatch II Two-Hybrid System (Stratagene) was used to detect interactions between CmtR and Zur or CmtR and IdeR, as described previously (53). The positive co-transformants were selected on the selective screening medium plate containing 5 mm 3-amino-1,2,4-triazole (Stratagene), 8 g/ml streptomycin, 15 g/ml tetracycline, 34 g/ml chloramphenicol, and 50 g/ml kanamycin (Kan). The co-transformants containing pBT-LGF2 and pTRG-Gal11^p^ (Stratagene) were used as the positive controls, while those containing the empty vectors pBT and pTRG were used as the negative controls.

### SPR analysis

The interaction between CmtR and Zur was analyzed using a Biacore 3000 instrument (GE Healthcare) according to previously published procedures (53). The His-tagged CmtR proteins (200 nm) were immobilized onto NTA chips, and 125–1000 nm Zur proteins were passed over the NTA chips at a flow rate of 10 ml/min at 25°C. For negative controls, Zur was substituted with MabR (125–1000 nm) under the same experimental conditions. The proteins were diluted using running buffer (10 mm HEPES, pH 7.4, 150 mm NaCl, 50 mm EDTA, and 0.005% Biacore surfactant P20) to the indicated concentration. Each analysis was performed in triplicate. Several overlay plots were constructed to depict the interactions using BIAevaluation 3.1 software.

### Evaluation of mycobacterial growth

The growth patterns of *M. bovis* BCG strains were evaluated using modified versions of procedures described earlier (47). The recombinant strains were cultured in 7H9 medium supplemented with 30 g/ml Kan or supplemented with 50 ng/ml anhydrotetracycline hydrochloride for strains containing pMindD plasmids, and the cultures were incubated under shaking conditions at 160 rpm at 37°C. When the culture reached the mid-logarithmic phase, each culture was diluted (4:100) in 50 ml of fresh 7H9 broth containing the corresponding antibiotics mentioned above, and the cultures were divided into 10 equal volumes. Six of these were divided into two groups and separately treated with 0/0.75 mm H_2_O_2_ under shaking conditions at 160 rpm at 37°C. Next, 0.75 mm H_2_O_2_ was added to the medium at 0, 2, and 4 days to ensure that the medium contained significant levels of H_2_O_2_ throughout the duration of the experiment. The sensitivity of the recombinant strains to H_2_O_2_ stress was determined every 2 days. Serial dilutions of the samples were plated on 7H10 agar, and the number of cfu formed after 15–21 days was counted. Different WT and knockout strains were used during the experiments, and the concrete strains were indicated in the corresponding figure legend.

### Determination of metal ion content under H_2_O_2_ stress

Total zinc, iron, and manganese ion contents in the dried bacterial pellets were determined by ICP-OES (Varian, USA) according to a previously published procedure (54) with several modifications. Briefly, the *cmtR* and *esxH* recombinant strains were cultured in 300 ml of 7H9 medium under shaking conditions at 160 rpm at 37°C until the *A*_600_ value reached 0.5, after which the strains were treated with 5 mm H_2_O_2_ for 24 h. The harvested samples were washed twice in PBS containing 1 mm EDTA and 0.05% Tween 80, and were then washed only with PBS. The pellets were stored overnight at −80°C and dried using the Freeze Dryer machine (Thermo Fisher). The dried samples were weighed and acid-digested with HNO_3_ (trace metal grade) for 4 h at 80°C and overnight at 65°C. The digestion experiments were terminated by adding one-eighth volume of 30% (v/v) H_2_O_2_ at 1:10 dilution with water. The supernatants were filtered by passing through 0.22-μm filters (Corning Inc.). The metal content in the digested samples was measured by ICP-OES.

### Assays for the survival of mycobacteria under H_2_O_2_ stress

Assays for the sensitivity of mycobacteria to H_2_O_2_ were conducted as described previously (55) with certain modifications. Briefly, the *cmtR*-deleted and WT *M. bovis* BCG strains were cultured in 100 ml of 2× Sauton's medium supplemented with 0.2% glycerol, 0.05% Tween 80, 30 g/ml Kan. When the *A*_600_ value reached 0.5, each culture was divided into 27 equal volumes, further divided into nine groups, and separately treated with the indicated concentrations of Zn^2+^, the Zn^2+^ chelator N, N, N′, N′-tetrakis(2-pyridylmethyl) ethylenediamine (TPEN) (Sigma), and H_2_O_2_ for 24 h. The cells were diluted and plated on 7H10 agar plates, and the cfu formed after 15-21 days were counted. The survival percentages were calculated based on cfu (with treatment)/cfu (without treatment) for each strain.

### Mice

WT female SPF C57BL/6 mice were purchased from Changsheng Bio (Liaoning, China). The mice weighed 16–18 g and were 6–8 weeks old and housed in a specific pathogen-free facility using standard humane animal husbandry protocols that were approved by the Research Ethics Committee of the College of Veterinary Medicine, Huazhong Agricultural University, Hubei, Wuhan, China (HZAUMO-2019-013).

### Preparation of BMDMs and infection

BMDMs were obtained by flushing the tibia and femurs of mice, as described previously (56). The cells were cultured in DMEM/F12 (HyClone, USA) supplemented with 10% FBS (Gibco, USA), 1% nonessential amino acid (NEAA, Gibco, USA), 1% Na-Pyrurate (Gibco, USA), 10% penicillin-streptomycin (Gibco, USA), and 30% L929 cell supernatant (containing macrophage colony-stimulating factor) in 5% CO_2_ for 3 days at 37°C, after which the well-mixed medium and L929 cell supernatant were added to the cultures for another 2 days. Mature BMDMs (5 × 10^5^/well) were seeded in microculture dishes (Thermo Fisher Scientific) overnight and were subsequently used for bacterial infection.

### Intracellular survival assays

The BMDMs and RAW264.7 cells were seeded in 24-well plates and cultured overnight. The cells were then infected with *M. bovis* BCG strains at a multiplicity of infection of 10, as described previously (56). At 4 hpi, the macrophages were washed thrice with PBS to remove extracellular bacteria and were then added to the well-mixed medium. After incubation for 1–48 h for RAW264.7 cells and for 2–30 h for BMDMs, the cells were lysed using 0.025% SDS and diluted for plating on 7H10 agar plates for counting cfu 15–21 days later. Penicillin/streptomycin was added to the medium, except during infections.

### Mouse infection

The female SPF C57BL/6 mice weighed 16–18 g and were 6–8 weeks old during the experimental period. *M. bovis* BCG strains were cultured till the *A*_600_ value reached 1.0, and were washed three times with PBS containing 0.05% Tween 80. Thirty-six mice were randomly divided into six groups (*n* = 6), and were intratracheally infected with 1 × 10^6^ cfu of WT/pMV261, △ *cmtR*/pMV261, and OE*-cmtR* strains separately, and data were analyzed as described previously (57).

### Statistical analysis

Statistical analyses of data were performed using unpaired two-tailed Student's *t* test with GraphPad Prism 7. Data are expressed in terms of mean ± S.D. *Asterisks* represent significant difference, *, *p* < 0.05; **, *p* < 0.01; ***, *p* < 0.001; and *ns*, not significant (*p* ≥ 0.05).

## Data availability

All data described are presented either within the article or in the supporting information.

## Supplementary Material

Supporting Information
