# Supplementary material for: A novel stress-inducible CmtR-ESX3-Zn2+ regulatory pathway essential for survival of Mycobacterium bovis under oxidative stress
Source: J Biol Chem. 2020 Oct 8;295(50):17083–99. doi: 10.1074/jbc.RA120.013017 (PMC7863910; doi:10.1074/jbc.RA120.013017)
Supplement: Supporting Information [file supp_295_50_17083__index.html]

A novel stress-inducible CmtR-ESX3-Zn2+ regulatory pathway essential for survival of Mycobacterium bovis under oxidative stress — A novel CmtR-triggered antioxidant pathway in mycobacteria — A novel stress-inducible CmtR-ESX3-Zn2+ regulatory pathway essential for survival of Mycobacterium bovis under oxidative stress — A novel CmtR-triggered antioxidant pathway in mycobacteria — Supporting Information 

# A novel stress-inducible CmtR-ESX3-Zn2+ regulatory pathway essential for survival of *Mycobacterium bovis* under oxidative stress

## Supporting Information

- Supporting Information (to be published online) - Supplementary information
