# Supplementary material for: A novel stress-inducible CmtR-ESX3-Zn2+ regulatory pathway essential for survival of Mycobacterium bovis under oxidative stress
Source: J Biol Chem. 2020 Oct 8;295(50):17083–99. doi: 10.1074/jbc.RA120.013017 (PMC7863910; doi:10.1074/jbc.RA120.013017)
Supplement: Supporting Information [file supp_RA120.013017_158797_3_supp_606053_qhkm28.pdf]

A novel stress-inducible CmtR-ESX3-Zn^{2+} regulatory pathway essential for survival of *Mycobacterium bovis* under oxidative stress

Xiaohui Li[1], Liu Chen[1], Jingjing Liao[1], Jiechen Hui[1],Weihui Li[2], Zheng-Guo He*[,1,2]

[1]College of Life Science and Technology, Huazhong Agricultural University, Wuhan 430070, China
[2]State Key Laboratory for Conservation and Utilization of Subtropical Agro-bioresources, College of Life Science and Technology, Guangxi University, Nanning 530004, China.

*To whom correspondence should be addressed: College of Life Science and Technology, Guangxi University, Nanning 530004, China.
Email: hezhengguo2019@163.com
Tel: +86-771-3225146, Fax: +86-771-3225146

Running title: A novel CmtR-triggered antioxidant pathway in mycobacteria

Keywords: *Mycobacterium bovis*; oxidative stress; ESX-3; zinc ion; CmtR

Supplemental Information

Figure S1. Physical map of the *cmtR* gene region and amino acid sequence alignment of CmtR in *M. tuberculosis* H37Rv.

Figure S2. Assays for evaluating the DNA-binding ability of CmtR and the effect of H_2O_2 on the intracellular DNA-binding activity of EthR in *M. bovis* BCG.

Figure S3. Assays for studying the effects of H_2O_2 on the DNA-binding ability of CmtR and its mutant variants.

Figure S4. Assays for studying the effect of CmtR (C24S) on the growth of *M. bovis* BCG under H_2O_2 stress.

Figure S5. The functional categories of the potential target genes of CmtR in *M. tuberculosis* H37Ra.

Figure S6. Assays for studying the effects of H_2O_2 on the expression of *esx-3* operon genes in *M. bovis* BCG strains.

Figure S7. Assays for studying the growth of *M. bovis* BCG strains in 7H9 medium.

Figure S8. Assays for studying the survival of *M. bovis* BCG strains under H_2O_2 stress.

Table S1. Primers used in this study.

Table S2. Strains and plasmids used in this study.

A

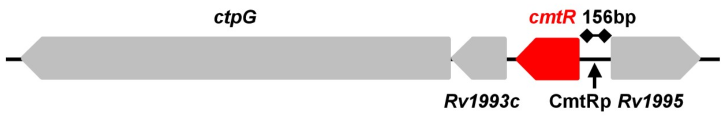

B

```
Mtu: Rv1994c      MLTCE------------MRESALARLGRALADPTRCRILVALLDGVCYPGQLAAHLGLTR  48
Mra: MRA_2010     MLTCE------------MRESALARLGRALADPTRCRILVALLDGVCYPGQLAAHLGLTR  48
Mbb: BCG_2011c    MLTCE------------MRESALARLGRALADPTRCRILVALLDGVCYPGQLAAHLGLTR  48
Msm: MSMEG_5603   -MQTA------------LHTDALARFGHALSDVTRTRILLSLNESPNYPADLAEQLGVSR  47
Mmi: MMAR_2139    -MPMKIADDAPPTASPREDLAGAVALFHSLSDPTRLAIARRLADGERRVVDLTRELGLPQ  59

Mtu: Rv1994c      SNVSNHLSCLRGCGLVVATYEGRQVRYALADSHLARALGELVQVVLA---VDTDQPCVAE  105
Mra: MRA_2010     SNVSNHLSCLRGCGLVVATYEGRQVRYALADSHLARALGELVQVVLA---VDTDQPCVAE  105
Mbb: BCG_2011c    SNVSNHLSCLRGCGLVVATYEGRQVRYALADSHLARALGELVQVVLA---VDTDQPCVAE  105
Msm: MSMEG_5603   QTLSNHLACLRGCGLVVAVPEGRRTRYELADARIGRALDDLMGLVLD---VDPECRCVGP  104
Mmi: MMAR_2139    STVSSHLACLRDCGLIAGRPEGRQVFYALAVPDLLDLFAAAETVLAATGNAVTLCPNYGT  119

Mtu: Rv1994c      RAASGEAVEMTGS--------------  118
Mra: MRA_2010     RAASGEAVEMTGS--------------  118
Mbb: BCG_2011c    RAASGEAVEMTGS--------------  118
Msm: MSMEG_5603   DGAVCGCG-------------------  112
Mmi: MMAR_2139    RPTRASASRRRGVKRAAPGVADWSPGR  146
```

Figure S1. Physical map of the *cmtR* gene region and amino acid sequence alignment of CmtR in *M. tuberculosis* H37Rv. (**A**) The *cmtR*(*Rv1994c*) operon genes in the *M. tuberculosis* H37Rv genome are shown. The CmtR-regulated region (*cmtRp*) are indicated by black arrows. (**B**) Amino acid sequence alignment (generated using ClustalW) of CmtR for the analysis of cysteine residue conservation among different mycobacterial species. The sequences shown are Rv1994c from *M. tuberculosis* H37Rv (Mtu), MRA_2010 from *M. tuberculosis* H37Ra (Mra), BCG_2011c from *M. bovis* BCG (Mbb), MSMEG_5603 from *Mycobacterium smegmatis* (Msm), and MMAR_2139 from *Mycobacterium marinum* (Mmi). The terms marked in red represent different cysteine residue sites in the sequence.

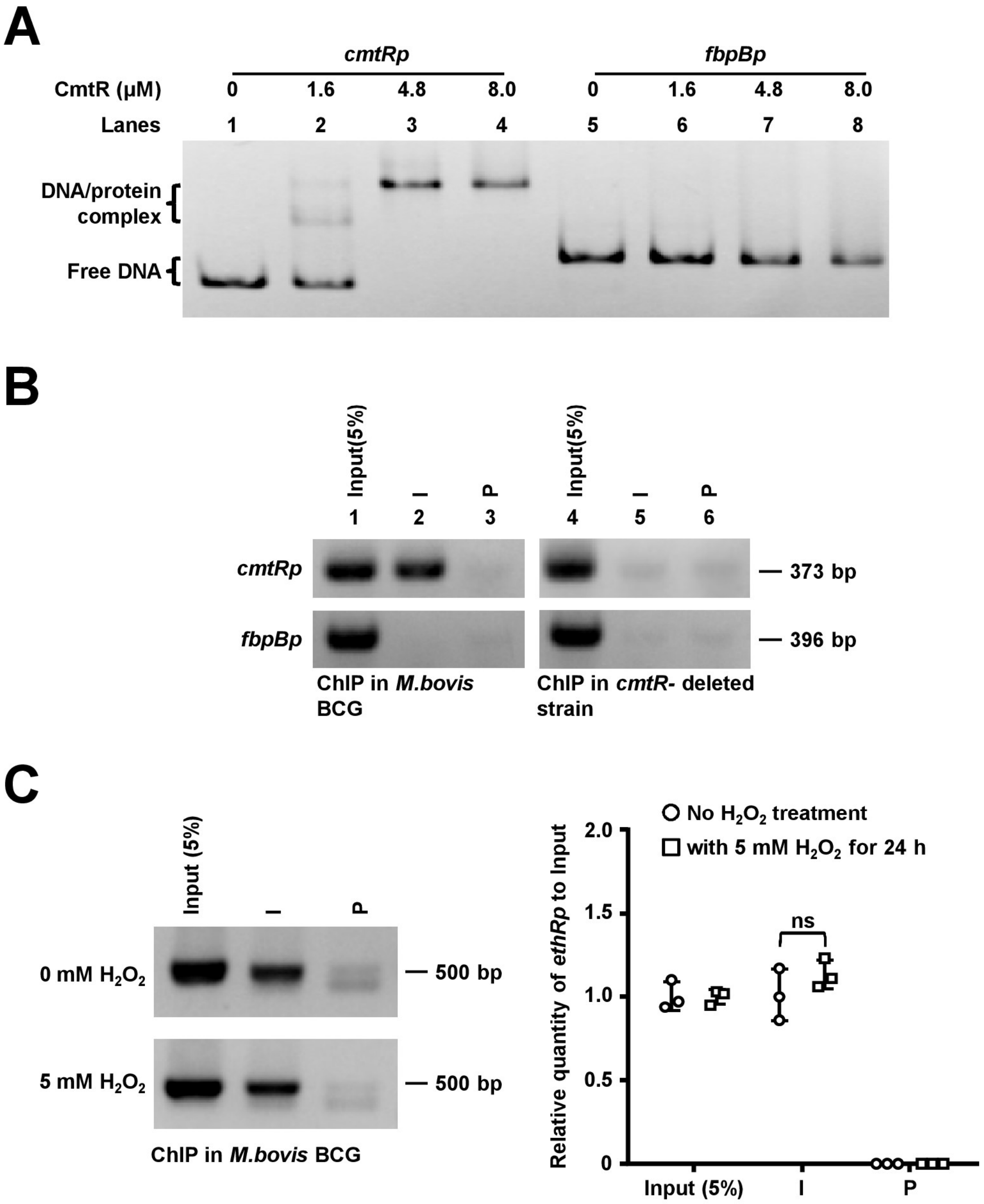

Figure S2. Assays for evaluating the DNA-binding ability of CmtR and the effect of H_2O_2 on the intracellular DNA-binding activity of EthR in *M. bovis* BCG. (**A**) EMSA. Either *cmtRp* promoter DNA (lanes 1-4) or *fbpBp* (lanes 5-8) were co-incubated with increasing quantities of CmtR, and the DNA-binding activities of the regulator were determined. (B) ChIP assays. ChIP using preimmune (P) or immune sera (I) against *cmtRp*. The mycobacterial promoter *fbpBp* was used as a negative control. The experiments were performed either in *M. bovis* BCG or in the *cmtR*-deleted strains. (C) Quantitative ChIP assays for evaluating the effect of H_2O_2 on the intracellular DNA-binding activity of EthR in *M. bovis* BCG. The Input (5%) indicates that the supernatant of the culture containing disrupted cells was diluted to 5% and used as the template for PCR. ChIP using P or I sera against EthR. Quantification was performed using qPCR (the right panel). Error bars represent the SD from three independent experiments. The *P*-values of the data were calculated by two-tailed Student's *t*-test using GraphPad Prism 7. Asterisks represent significant

difference (ns, not significant, two-tailed Student's *t*-test) between two groups.

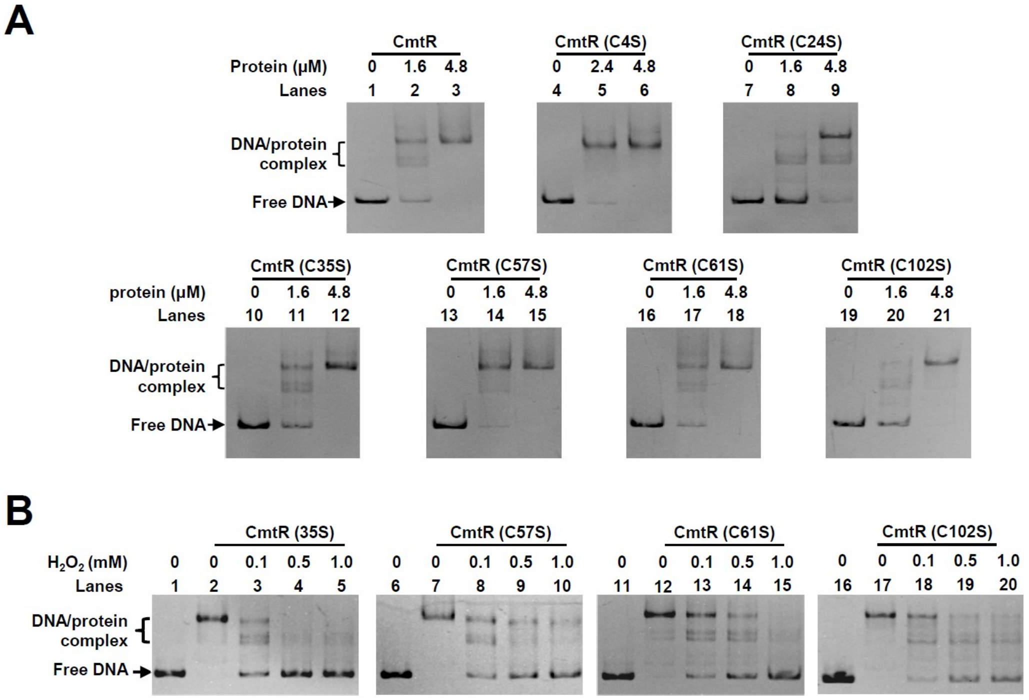

Figure S3. Assays for studying the effects of H_2O_2 on the DNA-binding ability of CmtR and its mutant variants. (**A**) EMSA for studying the DNA-binding ability of CmtR and its mutant variants. CmtR and its mutant variants were co-incubated with the *cmtRp* DNA substrate; increasing concentrations (1.6-4.8 μM) of the proteins are indicated at the top of the panels. (**B**) EMSA for studying the effects of H_2O_2 on the DNA-binding activity of CmtR mutant proteins. The protein concentration was 3.2 μM. The concentration (0.1-1 mM) of H_2O_2 is indicated at the top of the panels. The protein/DNA complexes are indicated by arrows on the left side of the panels.

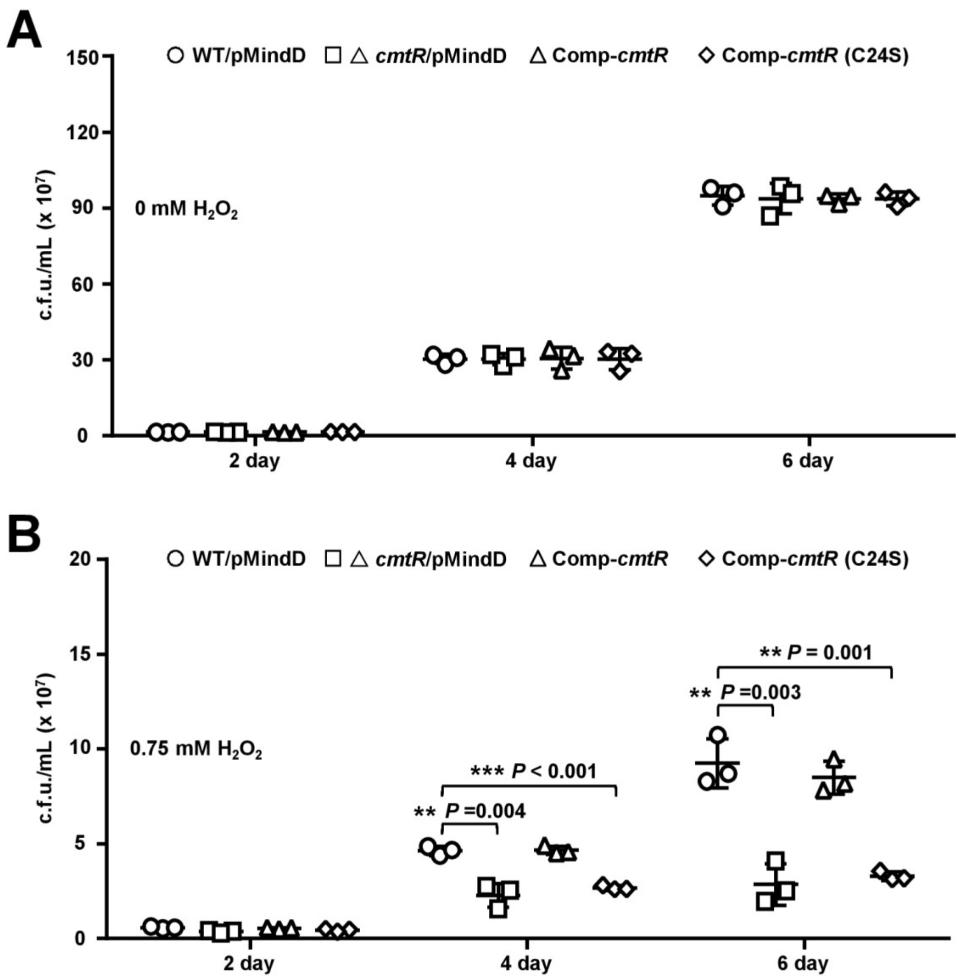

Figure S4. Assays for studying the effect of CmtR (C24S) on the growth of *M. bovis* BCG under H_2O_2 stress. Different mycobacterial strains were cultured in 7H9 medium (**A**) and the medium supplemented with 0.75 mM H_2O_2 (**B**). WT/pMindD represents the BCG/pMindD strain; △ *cmtR*/pMindD represents the BCG *cmtR*::*hyg*/pMindD strain; Comp-*cmtR* represents the BCG *cmtR*::*hyg*/ pMindD-*cmtR* strain; Comp-*cmtR* (C24S) represents the BCG *cmtR*::*hyg*/pMindD-*cmtR* (C24S) strain. Error bars represent the SD from three independent experiments. The *P*-values of the data were calculated by unpaired two-tailed Student's *t*-test using GraphPad Prism 7. Asterisks represent significant difference (**, $P<0.01$; ***, $P<0.001$, two-tailed Student's *t*-test) between two groups.

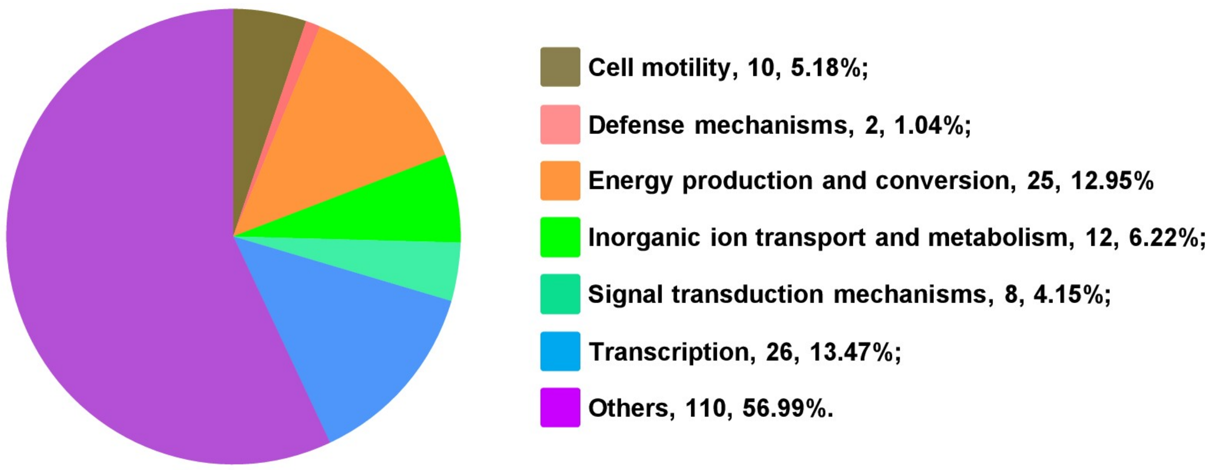

Figure S5. The functional categories of the potential target genes of CmtR in *M. tuberculosis* H37Ra. A functional classification of differentially expressed genes between wild-type and *cmtR*-deleted *M. tuberculosis* H37Ra strains in the context of Cluster of Orthologous Groups categories.

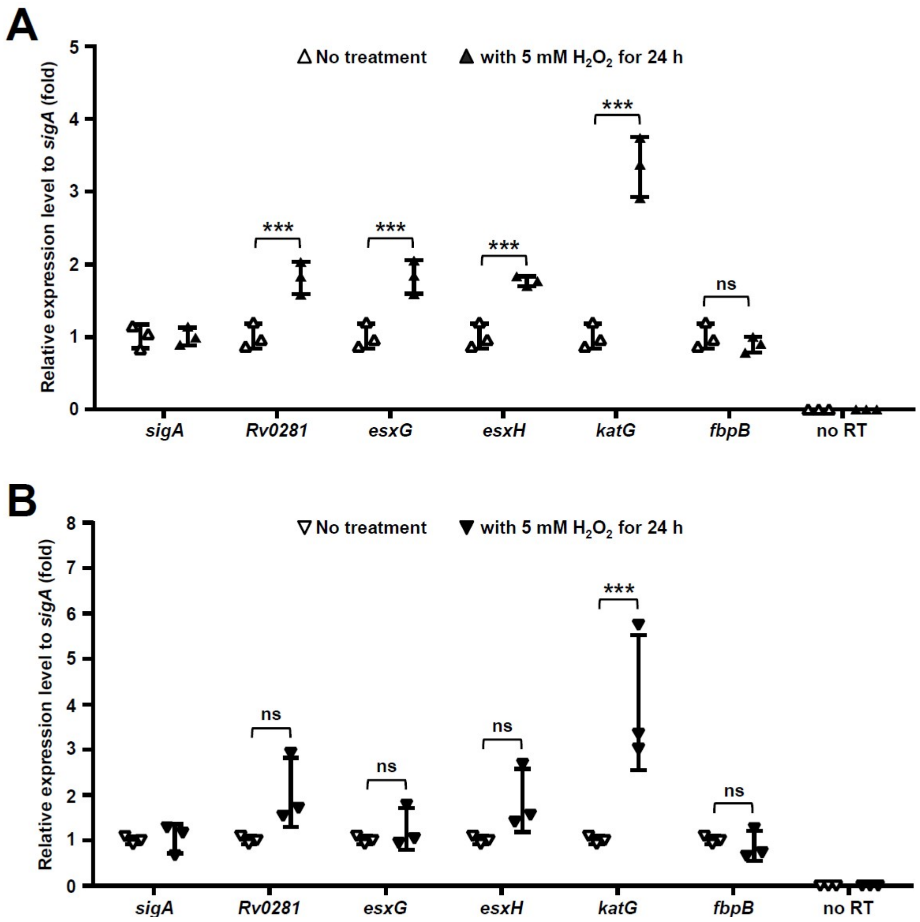

Figure S6. Assays for studying the effects of H_2O_2 on the expression of *esx-3* operon genes in *M. bovis* BCG strains. (**A** and **B**) qRT-PCR assays for studying the expression patterns of different genes (*Rv0281*, *esxG*, *esxH*, and *cmtR*) in *M. bovis* BCG WT (**A**) or *cmtR*-deleted strains (**B**) under treatment with indicated concentrations (0/5 mM) of H_2O_2 for 24 h. *KatG* and *fbpB* were used as control genes. Relative expression levels of the genes were normalized to those of *sigA*, which was used as an invariant transcript control using the $2^{-\Delta\Delta Ct}$ method. Error bars represent the SD from three independent experiments. The *P*-values of the data were calculated by unpaired two-tailed Student's *t*-test using GraphPad Prism 7. Asterisks represent significant difference (***, $P<0.001$; ns, not significant, two-tailed Student's *t*-test) between two groups.

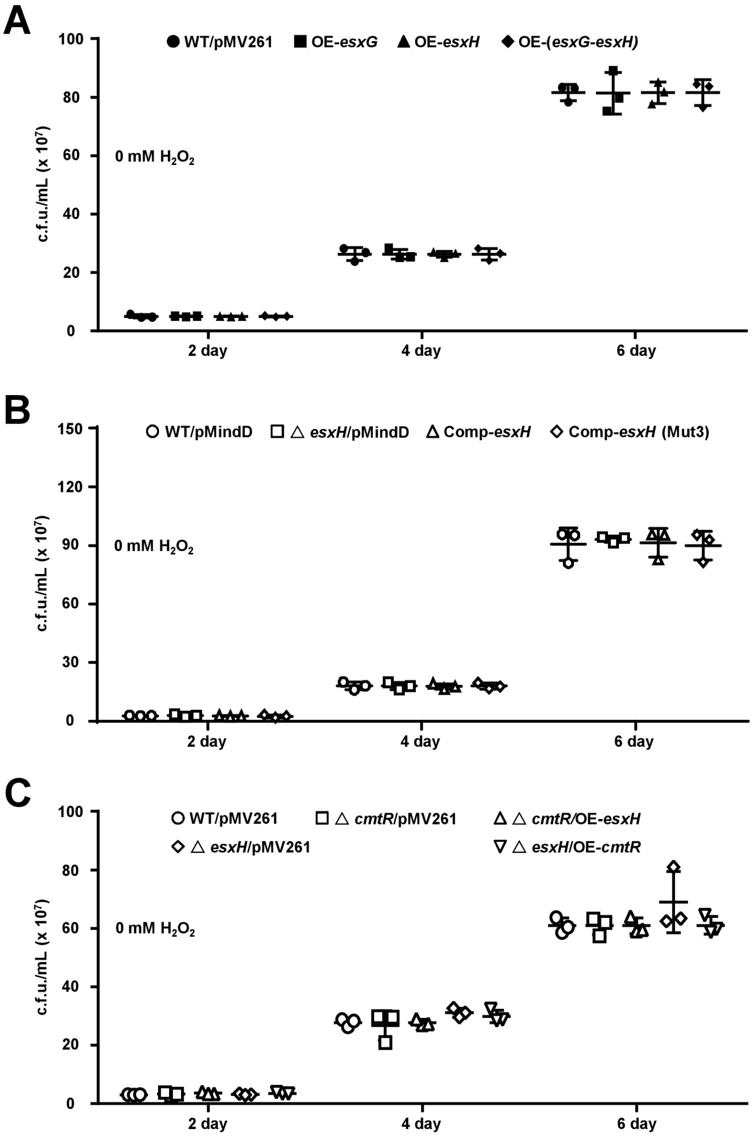

Figure S7. Assays for studying the growth of *M. bovis* BCG strains in 7H9 medium. (**A-C**) The recombinant mycobacterial strains were cultured in 7H9 medium for 6 days, and the bacterial counts were determined every 2 days. (**A**) WT/pMV261 represents the BCG/pMV261 strain; OE-*esxG*, OE-*esxH*, and OE-(*esxG-esxH*) represent the BCG/pMV261-*esxG*, BCG/pMV261-*esxH*, and BCG/pMV261-(*esxG-esxH*) strains, respectively; (**B**) WT/pMindD represents the BCG/pMindD strain; △ *esxH*/pMindD represents the BCG *esxH*::*hyg*/pMindD strain; Comp-*esxH* represents the BCG *esxH*::*hyg*/ pMindD-*esxH* strain; Comp-*esxH*-Mut3 represents the BCG *esxH*::*hyg*/pMindD-*esxH* (Mut3) (Mut3: H14A, H70A, H76A) strain. (**C**) WT/pMV261 represents the BCG/pMV261 strain; △ *cmtR*/pMV261 represents the BCG *cmtR*::*hyg*/pMV261 strain; △ *cmtR*/ OE-*esxH* represents the BCG *cmtR*::*hyg*/pMV261-*esxH* strain; △ *esxH*/pMV261 represents the BCG *esxH*::*hyg*/pMV261 strain; △ *esxH*/OE-*cmtR* represents the BCG *esxH*::*hyg*/pMV261-*cmtR* strain.

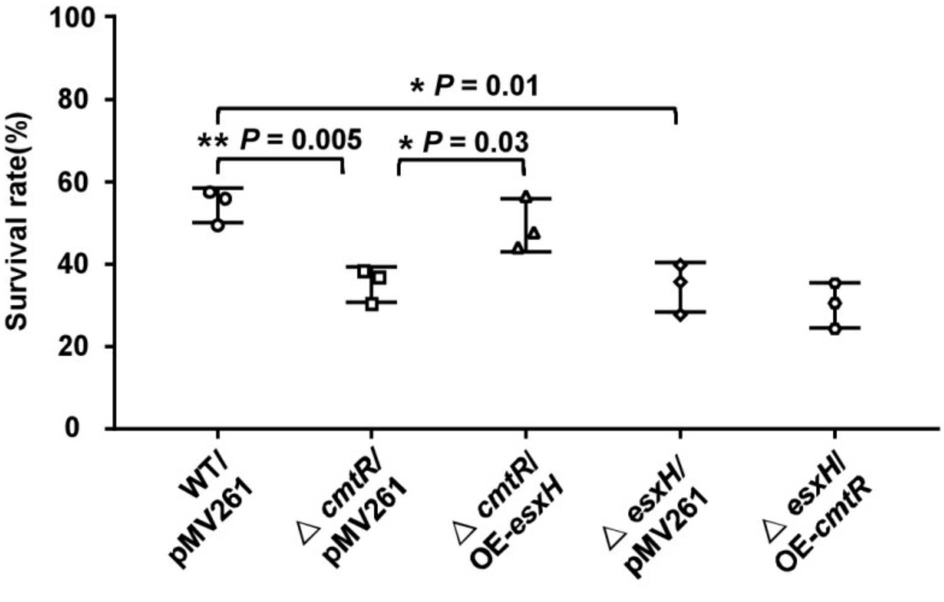

Figure S8. Assays for studying the survival of *M. bovis* BCG strains under H_2O_2 stress. The recombinant strains mentioned in the legend for Figure S7 C were cultured in Sauton's medium supplemented with 3 mM H_2O_2 for 24 h till an OD_{600} value of 0.5 was achieved, and the bacterial counts were determined. The survival rates were calculated in terms of CFU (with H_2O_2)/CFU (without H_2O_2) for each strain. Error bars represent the SD from three independent experiments. The *P*-values of the data were calculated by unpaired two-tailed Student's *t*-test using GraphPad Prism 7. Asterisks represent significant difference (*, $P<0.05$; ***, $P<0.001$, two-tailed Student's *t*-test) between two groups.

Table S1. Primers used in this study

Usage	Construct	Primer name	Sequence (from 5' to 3')
Cloning and expression (pMV261 or pET-28a)	CmtR	Rv1994cf	CCACGAATTCGTATGCTGACGTGTGAGATGCG
		Rv1994cr	CCGATCTAGATCTCAGCTACCTGTCATCTCGA
Cloning and expression	IdeR	Rv2711f	CCTGGAATTCCCATGAACGAGTTGGTTGATAC
		Rv2711r	TCGTGCTCTAGATCAGACTTTCTCGACCTTGA
Cloning and expression (pET-28a-Sumo)	CmtR	Rv1994cf	TTTGATGGATCCATGCTGACCTGCGAGATGCG
		Rv1994cr	GGGGGGAAAAGCTTTTAGCTGCCGGTCATTTCAACC
Cloning and expression (pET-28a-Sumo)	Zur	Rv2359f	AAAAATGGATCCATGAGTGCAGCCGGTGTC
		Rv2359r	CCAAAAGCTTTTAGCTCCGGCAGTCTGA
Cloning and expression (pET-28a-Sumo)	MabR	Rv2242f	ATTAGGATCCGTGAACGACAATCAGTTGGC
		Rv2242r	ATTAAAGCTTTCAGTGCGGCGTCGGATAGT
Cloning and expression	EsxG	Rv0287f	CTCCGAATTCGGATGAGCCTTTTGGATGCTCA
		Rv0287r	TACCTCTAGAGGTTAGAACCCGGTATAGGTCG
Cloning and expression	EsxH	Rv0288f	CCCTCCGAATTCGGATGTCGCAAATCATGTAC
		Rv0288r	TAACTCTAGAGTTAGCCGCCCCATTTGGC
site-directed mutagenesis	CmtR (C4S)	C4Sf	TTATGGATCCATGCTGACGTCTGAGATGCG
		C4Sr	CGCATCTCAGACGTCAGCAT
site-directed mutagenesis	CmtR (C24S)	C24Sf	CCGACGCGGTCCCGGATTCTG
		C24Sr	CAGAATCCGGGACCGCGTCGG
site-directed mutagenesis	CmtR (C35S)	C35Sf	GATGGCGTTTCCTATCCCGGCCAG
		C35Sr	CTGGCCGGGATAGGAAACGCCATC
site-directed mutagenesis	CmtR (C57S)	C57Sf	CATCTGTCGTCTTTGCGGGGCTG
		C57Sr	CAGCCCCGCAAAGACGACAGATG
site-directed mutagenesis	CmtR (C61S)	C61Sf	GTTTGCGGGGCTCCGGGCTGGTAG
		C61Sr	CTACCAGCCCGGAGCCCCGCAAAC
site-directed mutagenesis	CmtR (C102S)	C102Sf	CGACCAACCCTCTGTCGCCGAG
		C102Sr	CTCGGCGACAGAGGGTTGGTCG
site-directed mutagenesis	EsxH (H14A)	*EsxH* (H14A)f	ATGTTGGGTGCCGCCGGGGATAT
		EsxH (H14A)*r*	ATATCCCCGGCGGCACCCAACAT
site-directed mutagenesis	EsxH (H70A)	*EsxH* (H70A)f	TGCGGGCCTATGCTGCGATGTCCA
		EsxH (H70A)*r*	TGGACATCGCAGCATAGGCCCGCA
site-directed mutagenesis	EsxH (H76A)	*EsxH* (H76A)f	GTCCAGCACCGCTGAAGCCAACAC
		EsxH (H76A)*r*	GTGTTGGCTTCAGCGGTGCTGGAC
EMSA	Rv0280p	Rv0280pf	ACTGGCTAAATCCGTTGCCG
		Rv0280pr	AGCCGTAAACACCACAGAGG
EMSA	Rv0282p	Rv0282pf	CTACGCTGTTGAACGACTAC
		Rv0282pr	CATGCCAATGTCATCAC
ChIP/EMSA	fbpBp	Rv1886cpf	CATCGCACGCCACAAAC
		Rv1886cpr	GTGCCGATCATCAATCG
ChIP/EMSA	cmtRp	Rv1994cpf	GGGACTTCCTGTTCCGG

		Rv1994cpr	GATTCCCGCATCTCACAC
Clone to pMV261-lacZ	Rv0280p	Rv0280pLf	GACGGAATTCACTGGCTAAATCCGTTGCCG
		Rv0280pLr	GACGTCTAGAAGCCGTAAACACCACAGAGG
Clone to pMV261-lacZ	Rv0282p	Rv0282pLf	GAGGGAATTCCTACGCTGTTGAACGACTAC
		Rv0282pLr	GGGTGTCTAGACATGCCAATGTCATCAC
Clone to pMV261-lacZ	fbpBp	Rv1886cpLf	GATGGAATTCCATCGCACGCCACAAAC
		Rv1886cpLr	GCACTCTAGAGTGCCGATCATCAATCG
Clone to pMV261-lacZ	cmtRp	Rv1994cpLf	TTTCGAATTCGGGACTTCCTGTTCCGG
		Rv1994cpLr	GGTCTCTAGAGATTCCCGCATCTCACAC
Clone to pMV261-lacZ	LacZ	lacZf	TTCAAGCTTATGAGGATGAGGGAAGCAAG
		lacZr	ATGCGCTAGCTTATTTTTGACACCAGACCA
knock out of *cmtR*	CmtR	BCG_2011cUPf	TCACACTTAATTAAGTGTACTACCGACGTTAGCCAG
		BCG_2011cUPr	GGCGACTAGTAGATCAAATAGTACACC
knock out of *cmtR*	CmtR	BCG_2011cDNf	CCCGAAGCTTGCAGCAGACGACACGACTTGTG
		BCG_2011cDNr	AAACGCTAGCGCGATGGTCATCAGGGTGC
knock out of *esxH*	EsxH	BCG_0328UPf	CCCGACTTAATTAAGTCGCGGTGGTATCAAGG
		BCG_0328UPr	GGTGACTAGTCATCACAAGTCCTCTCG
knock out of *esxH*	EsxH	BCG_0328DNf	TTAGAAGCTTCTAGCTCGCGCTACATGG
		BCG_0328DNr	TTATTTTCGCTAGCGCAAACGGTGTCCCAGG
qRT-PCR	SigA	RT-sigAf	TCGCGCCTACCTCAAACAG
		RT-sigAr	CGTACAGGCCAGCCTCGAT
qRT-PCR	CmtR	RT-CmtRf	TGCTGGATGGCGTTTGCT
		RT-CmtRr	GGCCCTCATAGGTTGCGACTA
qRT-PCR	FbpB	RT-fbpBf	CGCGACATCAAGGTTCAGTT
		RT-fbpBr	CCGGCATGACTATCGACAGT
qRT-PCR	Rv0281	RT-Rv0281f	GGGCTGCTGATCTATCTCCC
		RT-Rv0281r	TCGCTCGTTGTAGACCAGTT
qRT-PCR	EsxG	RT-esxGf	CGGCTCAGGCGTTTCAC
		RT-esxGr	CCGCCGCCACAAACC
qRT-PCR	EsxH	RT-esxHf	CAGGCCGCGTTGCA
		RT-esxHr	CTGCCACGCCTGATACGT
qRT-PCR	KatG	RT-katGf	CCGAGATTGCCAGCCTTAA
		RT-katGr	GTTGACCTCCCACCCGACT

Notes: Restriction enzyme sites are underlined.

Table S2. Strains and plasmids used in this study

Plasmid or Strain	Relevant genotype or feature	Source or reference
Plasmid		
pET28a	Kan^r, lacZ operon, T7 promotor, His-Tag	Novagen
pET28a-*cmtR*	*cmtR* inserted in *EcoR*I-*Xba*I of pET28a	This study
pET28a-*cmtR*(C4S)	*cmtR* (C4S) inserted in *EcoR*I-*Xba*I of pET28a	This study
pET28a-*cmtR*(C24S)	*cmtR* (C24S) inserted in *EcoR*I-*Xba*I of pET28a	This study
pET28a-*cmtR*(C35S)	*cmtR* (C35S) *inserted in EcoR*I-*Xba*I of pET28a	This study
pET28a-*cmtR*(C57S)	*cmtR* (C57S) *inserted in EcoR*I-*Xba*I of pET28a	This study
pET28a-*cmtR*(C61S)	*cmtR* (C61S) inserted in *EcoR*I-*Xba*I of pET28a	This study
pET28a-*cmtR*(C102S)	*cmtR* (C102S) inserted in *EcoR*I-*Xba*I of pET28a	This study
pET28a-*ideR*	*ideR* inserted in *EcoR*I-*Xba*I of pET28a	This study
pET28a-Sumo	Kan^r, lacZ operon, T7 promotor, His-Tag	Novagen
pET28a-Sumo-*cmtR*	*cmtR* inserted in *BamH*I-*HindII*I of pET28a-Sumo	This study
pET28a-Sumo-*mabR*	*mabR* inserted in *BamH*I-*HindII*I of pET28a-Sumo	This study
pET28a-Sumo-*zur*	*zur* inserted in *BamH*I-*HindII*I of pET28a-Sumo	This study
pMV261	Kan^r, pAL5000 replicon	This study
pMV261-*cmtR*	*cmtR* inserted in *EcoR*I-*Xba*I of pMV261	This study
pMV261-*esxG*	*esxG* inserted in *EcoR*I-*Xba*I of pMV261	This study
pMV261-*esxH*	*esxH* inserted in *EcoR*I-*Xba*I of pMV261	This study
pMV261-*esxG-esxH*	*esxG-esxH* inserted in *EcoR*I-*Xba*I of pMV261	This study
pMV261-*lacZ*	*lacZ* fused with null-promoter	
pMV261-hsp60-*lacZ*	hsp60 promoter fused with *lacZ*	This study
pMV261-Rv0280p-*lacZ*	Rv0280p promoter fused with *lacZ*	This study
pMV261-Rv0282p-*lacZ*	Rv0282p promoter fused with *lacZ*	This study
pMV261-fbpBp-*lacZ*	fbpBp promoter fused with *lacZ*	This study
pMV261-cmtRp-*lacZ*	cmtRp promoter fused with *lacZ*	This study
pMindD	Kan^r, tet^r, pAL5000 replicon	
pMindD-*cmtR*	*cmtR* inserted in *EcoR*I-*Xba*I of pMindD	This study
pMindD-*cmtR*(C24S)	*cmtR* (C24S) inserted in *EcoRI-XbaI* of pMindD	This study
pMindD-*esxH*	*esxH* inserted in *EcoR*I-*Xba*I of pMindD	This study
pMindD- *esxH* (Mut3)	*esxH* (H14A, H70A, H76A) inserted in *EcoRI-XbaI* of pMindD	This study
Strain		
E.coli		
DH5a	Host for plasmid construction	TaKaRa
BL21(λDE3)	Host for protein expression	TaKaRa
M.bovis BCG		
BCG/WT	M. bovis BCG wild-type	ATCC
BCG/*cmtR*::*hyg*	BCG with *cmtR* replaced by *hyg*	This study
BCG/*esxH*::*hyg*	BCG with *esxH* replaced by *hyg*	This study
BCG/pMV261	BCG with pMV261	This study

BCG *cmtR*::hyg/pMV261	*cmtR* replaced by *hyg* in BCG, *cmtR*-deleted strain with pMV261	This study
BCG *cmtR*::hyg/pMV261-*esxH*	*cmtR* replaced by *hyg* in BCG, *cmtR*-deleted strain with pMV261-*esxH*	This study
BCG *esxH*::hyg/pMV261	*esxH* replaced by *hyg* in BCG, *esxH*-deleted strain with pMV261	This study
BCG *esxH*::hyg/pMV261-*cmtR*	*esxH* replaced by *hyg* in BCG, *esxH*-deleted strain with pMV261-*cmtR*	This study
BCG/pMV261-*cmtR*	*cmtR*-overexpression Strain	This study
BCG/pMV261-*esxG*	*esxG*-overexpression Strain	This study
BCG/pMV261-*esxH*	*esxH*-overexpression Strain	This study
BCG/pMV261-*esxG*-*esxH*	*esxG*-*esxH*-overexpression Strain	This study
BCG/pMinD	BCG with pMinD	This study
BCG *cmtR*::hyg/pMinD	*cmtR* replaced by *hyg* in BCG, *cmtR*-deleted strain with pMinD	This study
BCG *cmtR*::hyg/pMinD-*cmtR*	*cmtR* replaced by *hyg* in BCG, *cmtR*-deleted strain with pMinD-*cmtR*	This study
BCG *cmtR*::hyg/pMinD-*cmtR*(C24S)	*cmtR* replaced by *hyg* in BCG, *cmtR*-deleted strain with pMinD-*cmtR* (C24S)	This study
BCG *esxH*::hyg/pMinD	*esxH* replaced by *hyg* in BCG, *esxH*-deleted strain with pMinD	This study
BCG *esxH*::hyg/pMinD-*esxH*	*esxH* replaced by *hyg* in BCG, *esxH*-deleted strain with pMinD-*esxH*	This study
BCG *esxH*::hyg/pMinD-*esxH*(Mut3)	*esxH* replaced by *hyg* in BCG, *esxH*-deleted strain with pMinD-*esxH* (H14A, H70A, H76A)	This study
BCG/pMVL1	BCG with pMV261-*lacZ*, negative control of β- galactosidase assays	This study
BCG/pMVL2	BCG with pMV261-hsp60-*lacZ*, positive control of β-galactosidase assays	This study
BCG/pMVL3	BCG with pMV261-Rv0280p-*lacZ*	This study
BCG/pMVL4	BCG with pMV261-Rv0282p-*lacZ*	This study
BCG/pMVL5	BCG with pMV261-fbpBp-*lacZ*	This study
BCG/pMVL6	BCG with pMV261-cmtRp-*lacZ*	This study
BCG *cmtR*::*hyg*/pMVL1	*cmtR* replaced by hyg in BCG, *cmtR*-deleted strain with pMV261-*lacZ*, negative control of β- galactosidase assays	This study
BCG *cmtR*::*hyg*/pMVL2	*cmtR* replaced by hyg in BCG, *cmtR*-deleted strain with pMV261-hsp60-*lacZ*, positive control of β-galactosidase assays	This study
BCG *cmtR*::*hyg*/pMVL3	*cmtR* replaced by hyg in BCG, *cmtR*-deleted strain with pMV261-Rv0280p-*lacZ*	This study
BCG *cmtR*::*hyg*/pMVL4	*cmtR* replaced by hyg in BCG, *cmtR*-deleted strain with pMV261-Rv0282p-*lacZ*	This study
BCG *cmtR*::*hyg*/pMVL5	*cmtR* replaced by hyg in BCG, *cmtR*-deleted strain with pMV261-fbpBp-*lacZ*	This study
BCG *cmtR*::*hyg*/pMVL6	*cmtR* replaced by hyg in BCG, *cmtR*-deleted strain with pMV261-cmtRp-*lacZ*	This study
M. tuberculosis H37Ra		
H37Ra/WT	Wild-type *M.bovis* H37Ra	ATCC
H37Ra/*cmtR*::*hyg*	H37Ra with *cmtR* replaced by *hyg*	This study
